# Proximity Ligation Assay Detection of Protein–DNA Interactions—Is There a Link between Heme Oxygenase-1 and G-quadruplexes?

**DOI:** 10.3390/antiox10010094

**Published:** 2021-01-12

**Authors:** Wojciech Krzeptowski, Patryk Chudy, Grzegorz Sokołowski, Monika Żukowska, Anna Kusienicka, Agnieszka Seretny, Agata Kalita, Alicja Czmoczek, Jakub Gubała, Sonia Baran, Damian Klóska, Mateusz Jeż, Jacek Stępniewski, Krzysztof Szade, Agata Szade, Anna Grochot-Przęczek, Alicja Józkowicz, Witold N. Nowak

**Affiliations:** 1 Department of Medical Biotechnology, Faculty of Biochemistry, Biophysics and Biotechnology, Jagiellonian University, 30387 Kraków, Poland; wojciech.krzeptowski@uj.edu.pl (W.K.); patryk.chudy@doctoral.uj.edu.pl (P.C.); gsokolowski007@gmail.com (G.S.); monika.zukowska@doctoral.uj.edu.pl (M.Ż.); anna.kusienicka@doctoral.uj.edu.pl (A.K.); aga.seretny@gmail.com (A.S.); alicja.czmoczek@gmail.com (A.C.); 326549@uwr.edu.pl (J.G.); or sonia.baran@student.uj.edu.pl (S.B.); damian.kloska@doctoral.uj.edu.pl (D.K.); mateusz.jez@doctoral.uj.edu.pl (M.J.); jacek.stepniewski@uj.edu.pl (J.S.); krzysztof.szade@uj.edu.pl (K.S.); agata.szade@uj.edu.pl (A.S.); anna.grochot-przeczek@uj.edu.pl (A.G.-P.); alicja.jozkowicz@uj.edu.pl (A.J.); 2 Department of General Biochemistry, Faculty of Biochemistry, Biophysics and Biotechnology, Jagiellonian University, 30387 Kraków, Poland; agata.kalita@alumni.uj.edu.pl

**Keywords:** heme oxygenase-1, heme, G-quadruplex, proximity ligation assay

## Abstract

G-quadruplexes (G4) are stacked nucleic acid structures that are stabilized by heme. In cells, they affect DNA replication and gene transcription. They are unwound by several helicases but the composition of the repair complex and its heme sensitivity are unclear. We found that the accumulation of G-quadruplexes is affected by heme oxygenase-1 (*Hmox1*) expression, but in a cell-type-specific manner: hematopoietic stem cells (HSCs) from *Hmox1^−/−^* mice have upregulated expressions of G4-unwinding helicases (e.g., *Brip1*, *Pif1*) and show weaker staining for G-quadruplexes, whereas *Hmox1*-deficient murine induced pluripotent stem cells (iPSCs), despite the upregulation of helicases, have more G-quadruplexes, especially after exposure to exogenous heme. Using iPSCs expressing only nuclear or only cytoplasmic forms of Hmox1, we found that nuclear localization promotes G4 removal. We demonstrated that the proximity ligation assay (PLA) can detect cellular co-localization of G-quadruplexes with helicases, as well as with HMOX1, suggesting the potential role of HMOX1 in G4 modifications. However, this colocalization does not mean a direct interaction was detectable using the immunoprecipitation assay. Therefore, we concluded that HMOX1 influences G4 accumulation, but rather as one of the proteins regulating the heme availability, not as a rate-limiting factor. It is noteworthy that cellular G4–protein colocalizations can be quantitatively analyzed using PLA, even in rare cells.

## 1. Introduction

The past two decades have witnessed a significant growth of research interest in the G-quadruplexes (G4). These non-canonical structures (non-B form) of DNA, which were also described for RNA, are formed by guanine-rich sequences and comprise two or more self-stacking G-quartets (made by four guanines held in a plane by Hoogsteen hydrogen bonding) and are stabilized by monovalent cations [1]. Computational analysis of the human genome showed that hundreds of thousands of sequences could potentially form G4 structures [2,3,4,5]. However, the majority of such sequences seem to not form stable quadruplexes in vitro [6]. Nevertheless, mounting evidence showed that the localization of DNA–G4 is not distributed randomly in the genome because the presence of those structures has been identified in biologically functional regions. G-quadruplexes have been found to form in G-rich telomeres [7], in gene promoters [8], or origins of replication [9]. Finally, computational approaches allowed for mapping putative G-quadruplex-forming sequences that are also within RNA [10]. Taken together, G4 structures are believed to potentially influence DNA replication, gene transcription, and translation. Thus, they could provide an additional element in the regulation of various biological functions. Very recently, the importance of G-quadruplexes in the promoter sequence for regulating the expression of *c-Myc* in prostate cancer cells has been directly demonstrated [11].

With the wide variety of spectroscopic methods that are available, including nuclear magnetic resonance, X-ray and circular dichroism, or Raman spectroscopy, the structure and formation of several types of G4 have been described in buffers in vitro [12,13,14]. Nevertheless, to understand G-quadruplexes’ biological relevance, it is crucial to study these structures in cells in vivo. Two main approaches have been used for investigating the existence of G4 in cells. The first strategy utilizes small quadruplex-binding ligands [15,16], while the second is based on antibodies recognizing G-quadruplexes [17,18]. Both methods were successfully applied for the visualization of cellular G4.

Importantly, emerging pieces of evidence suggest that the formation of G-quadruplexes is regulated through interactions with different proteins. Particular attention has been paid to the helicases, which are enzymes involved in resolving G4. Among them, the best characterized are BLM (Bloom syndrome RecQ like helicase), BRIP1 (BRCA1 interacting protein C-terminal helicase-1, also known as FANCJ), PIF1 (PIF1 5′-to-3′ DNA helicase), and WRN (Werner syndrome RecQ like helicase) [19]. PIF1 is a helicase that is active in the nucleus (mainly at telomeres) and in mitochondria. It binds to G4, especially in the S phase [20,21]. BRIP1 forms a complex with BRCA1 and shows a greater affinity for G4 structures than for single-stranded or double-stranded DNA [22]. Both PIF1 and BRIP1 play an important role in the suppression of DNA instability at G4 motifs [20,22,23]. Besides helicases, a wide range of G4-binding proteins have also been identified so far (for a review, see reference [23]). The majority of evidence comes from in vitro studies, yet far less is known about their role in G4 unwinding or stabilization in cells.

In general, G4 toxicity stems from replication stress. During DNA synthesis, the replication forks can stall as a result of encounters between the replication complex and template modifications, such as the presence of G-quadruplexes. These stalled forks are a major source of genome instability [24]. An important mechanism that contributes to DNA damage tolerance is a direct bypass of template lesions via translesion DNA synthesis (TLS), which is mediated mainly by polymerase theta and encoded by *Polq* gene [20]. Furthermore, BRIP1 helicase is particularly active in TLS [22]. Interestingly, administration of the G4 stabilizing small molecule compounds slows down the replication and stops the replication forks [24].

One of the G4-stabilizing ligands is heme a ubiquitous cellular cofactor, known to control gene expression by regulating the activity of heme-dependent transcription activators or repressors [25]. A large fraction of cellular heme is associated with hemoproteins and remains exchange inert. A labile heme pool, which is available for heme signaling, is far less abundant and buffered at a concentration of below 1 μmol/L [26]. The labile fraction may increase after extracellular heme overload, enhanced heme synthesis, accelerated hemoprotein breakdown under oxidative conditions, or impaired heme degradation [25,27]. Free heme excess is known to enhance the generation of reactive oxygen species (ROS) and induces the oxidative stress that may cause damage primarily to lipid membranes, but also to proteins and nucleic acids [25]. Plenty of physicochemical studies showed that ferrous and ferric heme (Fe(II)-protoporphyrin IX and Fe(III)-protoporphyrin IX) binds tightly to various RNA and especially DNA G-quadruplexes [28,29,30,31,32]. Intramolecular parallel G4–heme structures or mixed-type G4–heme hybrids show significant oxidative activity (both one-electron and two-electron oxidation), with kinetics that is comparable to those of heme-utilizing protein enzymes, including peroxidases, peroxygenases, and monooxygenases [29,30,33]. One can suppose that the oxidative activity of G4–heme complexes may imply a potential mechanism for heme-mediated DNA oxidation. The availability of free heme depends on its cellular uptake, synthesis, and degradation. The latter process is directly regulated by heme oxygenases, namely, constitutively expressed *Hmox2* and transcriptionally induced *Hmox1*.

Heme degradation products are important bioactive molecules and have been intensively studied by many teams [33,34]. Rather less attention has been paid to the consequences of controlling free heme itself. A recent report highlighted the role of the stabilization of G4 by heme in the regulation of gene expression, including genes involved in cell-cycle progression [11]. However, there are still no data on the effect of the heme-induced and heme-degrading HMOX1 on G4 accumulation in cells. Our aim was therefore to assess whether Hmox1 constitutes a part of the G4-processing pathway to facilitate the resolving of G4 structures through the removal of heme and whether *Hmox1* deficiency will enhance the accumulation of heme-stabilized G-quadruplexes.

In order to fill the gap in our knowledge of the role of protein partners in the maintenance of G-quadruplexes in vivo, we propose a proximity ligation assay (PLA) as a useful method in such studies. Initially, this technique was designed to detect the localization of specific proteins in cells/tissues and to observe the dynamics of interactions between proteins of interest [35,36]. Using a G4-specific antibody, we adapted PLA for the in-cell visualization of interactions between G4 structures and different proteins. To the best of our knowledge, we showed for the first time in human cells that BRIP1 protein is located in the vicinity of G-quadruplexes. We also detected HMOX1 as the next, not-yet-described protein that colocalizes with G4. Finally, through a combination of fluorescence-activated cell sorting and in situ PLA, we were able to confirm the existence of those interactions in murine rare hematopoietic stem cells.

## 2. Materials and Methods

### 2.1. Cell Lines and Cell Culture

Cells were cultured at 37 °C in a humidified 5% CO_2_ atmosphere. HEK293T cells were cultured in DMEM High-Glucose medium (Biowest, Nuaillé, France) containing 10% fetal bovine serum (FBS, EurX, Gdańsk, Poland) and antibiotics (100 IU/mL penicillin and 100 μg/mL streptomycin, Sigma-Aldrich, St. Louis, MO, USA). The cells were routinely cultured in six-well culture plates (Falcon, Corning, NY, USA) or were seeded in 24-well plates (Falcon) onto glass coverslips covered with a thin layer of Geltrex (0.1 mg/mL, Gibco, Waltham, MA, USA).

Mouse *Hmox1^+/+^*- and *Hmox1^−/−^*-induced pluripotent stem cells (iPSCs) were obtained from C57BL6×FVB *Hmox1^+/+^* and *Hmox1^−/−^* fibroblasts, as described before [37,38]. Mouse iPSCs were cultured in DMEM High-Glucose medium (Biowest) containing 20% fetal bovine serum (EurX), 1% Non-Essential Amino Acids (Life Technologies, Carlsbad, CA, USA), 0.1 mM β-mercaptoethanol (Life Technologies), 1000 U/mL leukemia inhibitory factor (LIF, Millipore, Burlington, MA, USA), and antibiotics (100 IU/mL penicillin and 100 μg/mL streptomycin, Sigma-Aldrich) on six-well plates coated with 1:100 Geltrex (Gibco) in DMEM/F12 (Gibco). iPSCs *Hmox1^−/−^* and *Hmox2^−/−^* were generated using pSpCas9(BB)-2A-Puro plasmid [39] with an sgRNA encoding insert (top: CACCGGCCTTCCGGTGTAGCTCCGT, bottom: AAACACGGAGCTACACCGGAAGGCC). The knockout of *Hmox2* was confirmed using Western blotting.

In some experiments, cells were stimulated for up to 4 h or 24 h using various doses of hemin (Frontier Scientific, Logan, UT, USA), including 2 µmol/L, 10 µmol/L, and 20 µmol/L concentrations.

### 2.2. Mice

Animal work was done in accordance with national and European legislations with breeders register no. 0078 and user registry no. 0053 (Ministry of Science and Higher Education, Warsaw, Poland). Mice were used only for tissue collection, and therefore there was no ethical committee approval required. For the isolation of hematopoietic stem cells or fibroblasts, we used C57BL6×FVB *Hmox1^+/+^* or *Hmox1^−/−^* mice bred in our facility. Mice were housed in individually ventilated cages in specific pathogen-free conditions and had unlimited access to food and water.

### 2.3. Isolation of Cells from the Bone Marrow

Mice were euthanized via CO_2_ inhalation. Bone marrow was isolated from femurs and tibia. Bones were crushed in a mortar and pestle in 2% FBS in PBS. Bone marrow was collected into tubes, filtered through a cell strainer (100 μm), and centrifuged at 670× *g* for 10 min at 4 °C. Next, the cell pellet was resuspended in an RBC lysis buffer (0.15 mol/L NH_4_Cl, 10 mmol/L KHCO_3_, 0.1 mmol/L EDTA) and incubated for 7 min at room temperature. After the dilution of the lysis buffer with 2% FBS in PBS, the cells were centrifuged and washed with PBS. Finally, the cell pellet was resuspended with 100 μL of 2% FBS in PBS.

### 2.4. Hmox1 Genotyping

Genomic DNA from iPSC lines or mouse tissues was isolated using a Genomic Mini kit (A&A Biotechnology, Gdynia, Poland) according to the manufacturer’s instruction. Genomic DNA fragments specific to wild-type or mutant (knock out) mice were amplified using a KAPA Mouse Genotyping kit (Sigma-Aldrich, St. Louis, MO, USA) and specific primers (Jackson Laboratory protocol no. 22816): oIMR8553 (common) GTACATGCTGGCTGGGTTCT, oIMR7415 (mutant) GCCAGAGGCCACTTGTGTAG, oIMR8554 (wild-type reverse) CCATTTCTCAGGCAAGAAGG. The products were then separated on a 2% agarose (EurX) gel.

### 2.5. Primary Antibodies

For cell sorting, the following antibodies were used: CD3 (#555275, BD Pharmingen, San Jose, CA, USA), Ter119 (#553673, BD Pharmingen), B220 (#553090, BD Pharmingen), CD11b (#553311, BD Pharmingen), Ly-6C and Ly-6G (#553128, BD Pharmingen) diluted 1:200, Sca-1 (#558162, BD Pharmingen), c-kit (#47-1171, Invitrogen, Waltham, MA, USA), CD150 (#115910, BioLegend, San Diego, CA, USA), and CD48 (#103422, BioLegend) diluted 1:50. For the immunocytochemistry and PLA experiments, the cells were incubated overnight at 4 °C with the following primary antibodies diluted 1:100: anti-G4 (mouse 1H6 clone, #MABE1126, Millipore), anti-G4 (goat 1H6 clone, #Ab00389-24.1, Absolute Antibody, Redcar, UK), anti-BRIP1 (#NBP1-31883, Novus Biologicals, Centennial, CO, USA), anti-Histone H3 (#4499, Cell Signaling Technology, Danvers, MA, USA), anti-HMOX1 (#ADI-SPA-896, Enzo Life Sciences, Villeurbanne, France), anti-KU70 (#sc-1487, Santa Cruz Biotechnology, Dallas, TX, USA), anti-KU80 (#ab80592, Abcam, Cambridge, UK), and anti-PIF1 (#PA5-37136, Invitrogen).

### 2.6. Fluorescence-Activated Cell Sorting (FACS)

The cell sorting was done on a MoFlo XDP cell sorter (Beckman Coulter, Brea, CA, USA). The populations used in the studies were defined as follows: cKit^+^Lin^−^Sca1^+^ (KLS) CD48^−^CD150^+^ HSC, KLS CD150^−^CD48^−^ MPP, and KLS^−^ CD48^+^CD150^+^ GMP. The cells were sorted directly on poly-L-lysine coated slides (Thermo Scientific, Waltham, MA, USA) according to the published protocol [40].

For the flow cytometry analysis of G4 in *Hmox1^−/−^* mice, we used 4–6-month-old C57BL6×FVB *Hmox1^−/−^* or *Hmox1^+/+^* mice. Bone marrow cells after the staining for HSC markers were then fixed with 1% paraformaldehyde (Biotium, Fremont, CA, USA) in PBS, then permeabilized with 0.1% Triton X-100 in PBS and incubated overnight with a 1:100 anti-G4 antibody. Then, the cells were washed and stained with 1:200 AlexaFluor 488 anti-goat antibody for 30 min. After the staining, the cells were washed and analyzed on a BD LSR Fortessa flow cytometer (Becton Dickinson, Franklin Lakes, NJ, USA).

### 2.7. Transfection

HEK293T cells were transfected using jetPRIME (Polypus Transfection, Illkirch-Graffenstaden, France). The G-quadruplex formed ckit87up oligos (AGGGAGGGCGCTGGGAGGAGGG) or dsDNA oligos (CCAGTTCGTAGTAACCC, GGGTTACTACGAACTGG, and CCAGTTCGTAGTAACCC) [41]. Briefly, 20 μmol/L oligonucleotides (Genomed, Warsaw, Poland) in 10 mmol/L Tris-HCl pH 8.0 with 100 mmol/L KCl were incubated for 5 min at 95 °C and left at room temperature to cool down. For transfection, we used 18 nmol/L of treated oligonucleotides.

For free heme detection, the cells were transfected with a 1 μg cytosolic or nuclear HS1 heme sensor plasmid [26] using PEI Max (Polysciences, Warrington, PA, USA) the day before the oligos transfection.

### 2.8. N-methylomesoporphyrin IX Staining

HEK293T cells transfected with either G-quadruplex-forming oligonucleotides or control dsDNA oligonucleotides were incubated with 10 μmol/L N-methylmesoporphyrin (Frontier Scientific). After 3 h of incubation, the cells were detached with TrypLE and their fluorescence was analyzed on a BD LSR Fortessa cytometer with a 630/30 filter (Becton Dickinson, Franklin Lakes, NJ, USA).

### 2.9. Immunocytochemistry

The cells grown on glass coverslips were fixed with ice-cold 80% methanol (POCh, Gliwice, Poland) at −20 °C for 10 min or 4% paraformaldehyde (PFA, Biotium, Fremont, CA, USA) at room temperature for 10 min and followed by two washes with PBS. Cells fixed in PFA were subsequently permeabilized in PBS with 0.2% Triton X-100 (PBS-Tx) at room temperature for 10 min. Next, cells were blocked in 10% normal donkey serum (Sigma-Aldrich) in PBS (methanol-fixed cells) or in 0.1% PBS-Tx (PFA-fixed cells) at room temperature for one hour. The cells were then incubated with primary antibodies diluted in PBS (methanol-fixed cells) or 0.1% PBS-Tx (PFA-fixed cells) with 1% donkey serum (Sigma-Aldrich). Blocking was followed by three PBS washes before incubating with the appropriate secondary antibodies that were diluted 1:500 in PBS (methanol-fixed cells) or 0.1% PBS-Tx (PFA-fixed cells) containing 1% donkey serum at room temperature for three hours. The following secondary antibodies from Molecular Probes (Invitrogen) were used: AlexaFluor 488 anti-rabbit IgG (#A21206), AlexaFluor 568 anti-mouse IgG (#A10037), and AlexaFluor 568 anti-goat IgG (#A11057). Finally, following washing with PBS and counterstaining the cell nuclei with 0.5 μg/mL DAPI (Sigma-Aldrich) for 10 min at room temperature, the coverslips were mounted in Fluorescence Mounting Medium (Dako, Santa Clara, CA, USA) and allowed to dry before imaging. Negative controls with the omission of primary antibodies were performed for each protein.

### 2.10. DNase and RNase Treatment

After fixation in methanol, coverslips were incubated with DNase I (25 μg/mL, Worthington Biochemical, Lakewood, NJ, USA) or RNase A (100 μg/mL, Invitrogen) for 45 min at 37 °C. Next, the cells were washed three times with PBS before further procedures.

### 2.11. In Situ PLA

For the in situ visualization of G4–protein or protein–protein interactions, we followed the Duolink PLA Fluorescence Protocol (Sigma-Aldrich) using the Duolink In Situ Detection Reagents Orange kit (#DUO92007, Sigma). Briefly, after fixation with 80% methanol (HEK293T cells) or after fixation with 4% PFA and permeabilization with 0.2% PBS-Tx at room temperature for 10 min (FACS sorted cells), the cells were blocked in a drop of Blocking Solution (Sigma-Aldrich) at 37 °C for one hour and incubated with primary antibodies that were diluted in Duolink Antibody Diluent (Sigma-Aldrich). Following washing in wash buffer A (10 mM Tris, 150 mM NaCl, and 0.05% Tween 20), cells were incubated with secondary antibodies conjugated with PLUS and MINUS probes (Sigma-Aldrich) for one hour at 37 °C. The following Duolink In Situ PLA secondary antibodies were used: Anti-Mouse PLUS (#DUO92001), Anti-Goat PLUS (#DUO92003), and Anti-Rabbit MINUS (#DUO92005). Next, the cells were again washed twice in wash buffer A and then incubated with the ligase (diluted 1:40 in ligation buffer) for 30 min at 37 °C. Following the next round of washing in wash buffer A, cells were incubated with the polymerase (diluted 1:80 in an amplification buffer) for 100 min at 37 °C. Finally, the cells were washed in wash buffer B (200 mmol/L Tris and 100 mmol/L NaCl), counterstained with DAPI (0.5 μg/mL, Sigma-Aldrich), mounted in Fluorescence Mounting Medium (Dako), and allowed to dry before imaging. Negative controls were performed using only one primary antibody or secondary antibodies only.

### 2.12. Confocal Microscopy

We performed scanning laser confocal microscopy with a Zeiss LSM 880 microscope (Zeiss, Oberkochen, Germany). For excitation, a diode laser (405 nm), an argon laser (488 nm), and a diode laser (561 nm) were used. Crosstalk between the channels was eliminated with sequential scanning. We imaged single optical sections using the Plan-Neofluar 40×1.30 Oil DIC M27, alpha Plan-Apochromat 63×/1.4 Oil DIC, or Plan-Apochromat 100×/1.46 Oil DIC objectives (all Zeiss).

### 2.13. Flow Cytometry PLA

For analysis of the G4–protein or protein–protein interactions using flow cytometry, we followed the Duolink PLA Flow Cytometry Protocol (Sigma-Aldrich) using a Duolink flowPLA Detection Kit—Green (#DUO94002, Sigma-Aldrich). Briefly, the cells from individual plates were trypsinized, collected into Eppendorf tubes, and centrifuged at 500× *g* for 5 min. Next, the cells were washed with 1% FBS in PBS, counted, and fixed in cold 80% methanol at −20 °C for 10 min. After two washes with PBS, the cells were blocked in Blocking Solution (Sigma-Aldrich) at 37 °C for one hour. Next, the cells were aliquoted in a 96-well V-bottom plate (100,000 cells per well) and then incubated with primary antibodies that were diluted in Duolink Antibody Diluent (Sigma-Aldrich). Following washing in wash buffer A (10 mmol/L Tris, 150 mmol/L NaCl, and 0.05% Tween 20), the cells were incubated with secondary antibodies conjugated with PLUS and MINUS probes (Sigma-Aldrich) for one hour at 37 °C. The following Duolink In Situ PLA secondary antibodies (Sigma-Aldrich) were used: Anti-Mouse PLUS (#DUO92001) and Anti-Rabbit MINUS (#DUO92005). Next, the cells were again washed twice in wash buffer A and then incubated with the ligase (diluted 1:40 in ligation buffer) for 30 min at 37 °C. Following the next round of washing in wash buffer A, the cells were incubated with the polymerase (diluted 1:80 in amplification buffer) for 100 min at 37 °C, then incubated in a detection buffer for 30 min at 37 °C. Finally, the cells were washed in wash buffer B (200 mmol/L Tris and 100 mmol/L NaCl), counterstained with DAPI (0.5 μg/mL, Sigma-Aldrich), and re-suspended with 200 μL of 2% FBS in PBS. The cells were analyzed with a BD LSR Fortessa (Becton Dickinson, Franklin Lakes, NJ, USA) and an Amnis Image Stream X System (Amnis, Luminex, Austin, TX, USA).

### 2.14. RNA-Seq

We reanalyzed our previously published data that are available in the BioProject database, accession no.: PRJNA562450 [42].

### 2.15. Reverse Transcription and Real-Time PCR

RNA was isolated using an RNeasy Mini Kit (Qiagen, Venlo, Netherlands) and reverse-transcribed with a QuantiTect Reverse Transcription Kit (Qiagen) with integrated gDNA removal. The gene expression was assessed on a StepOnePlus thermocycler (Applied Biosystems, Waltham, MA, USA) with real-time PCR using an SYBR Green JumpStart Taq ReadyMix (Sigma-Aldrich) and specific primers: *EEF2* For—GCG GTC AGC ACA ATG GCA TA, Rev—GAC ATC ACC AAG GGT GTG CAG; HMOX1 For—CAA CAA AGT GCA AGA TTC TG, Rev—TGC ATT CAC ATG GCA TAA AG; Hprt For—AGG GAT TTG AAA TCA CGT TTG, Rev—TTT ACT GGC AAC ATC AAC AG; B2m For—GTA TGC TAT CCA GAA AAC CC, Rev—CTG AAG GCA TAT CTG ACA TC; Pif1 For—GTT AGG CAG ATG TTC AGA TG, Rev—ATC ATC CTG ATG GGT ACA TAG; Brip1 For—AAG CTC ACA ACA TTG AAG AC, Rev—CCA ATT GAT GAG GTT ATA GCA C (all from Sigma-Aldrich).

### 2.16. Western Blotting

Electrophoresis was performed on a 10% polyacrylamide gel in a Mini-PROTEAN apparatus (Bio-Rad, Hercules, CA, USA) placed on ice. After the electrophoresis, the proteins were transferred to a PVDF membrane in a wet system, then blocked with 5% bovine albumin for 60 min at room temperature and incubated with a primary antibody solution (1:1000 rabbit anti-HMOX2, ADI-OSA-200, Enzo Life Sciences Villeurbanne, France) overnight at 4 °C, a TBST buffer (washing five times for 5 min with buffer exchange), a secondary antibody solution (60 min at room temperature), and a TBST buffer (washing three times for 5 min with buffer exchange). While using a substrate for HRP, chemiluminescence detection was performed on a ChemiDoc MP instrument (Bio-Rad).

### 2.17. Chromatin Immunoprecipitation (ChIP)

ChIP analysis was carried out according to the protocol ChIP-IT High Sensitivity (Active Motif, Carlsbad, CA, USA). Mouse *Hmox1^+/+^* and *Hmox2^+/+^* iPSCs were seeded in six-well plates and were cultured until they were approximately 90% confluent. To increase the Hmox1 levels, cells were stimulated for 4 h with 2 µmol/L hemin. An input step was made to check the correctness of the chromatin isolation (adding NaCl and heating at 100 °C steps were omitted). Immunoprecipitation was performed using an anti-Hmox1 antibody (#ADI-SPA-896-F, Enzo Life Sciences, Villeurbanne, France) and control rabbit IgG (#AB-105-C, R&D Systems, Minneapolis, MN, USA). Six micrograms of antibodies were used per sample. The final step was performed to check the potential DNA sequences bound by HO1. For the detection of potential DNA sequences bound to Hmox1 protein, an electrophoretic separation was performed on a 1% agarose gel with ethidium bromide.

### 2.18. Statistical Analysis

The data were analyzed with GraphPad Prism 9.0 software (GraphPad Software, San Diego, CA, USA). The type of test used is described in the figure legends. We considered a result to be statistically significant when *p* < 0.05.

## 3. Results

### 3.1. Hmox1^−/−^ Hematopoietic Stem Cells Enhanced the Expression of G-quadruplex Helicases and Had Fewer G-quadruplexes

**Figure 1:**
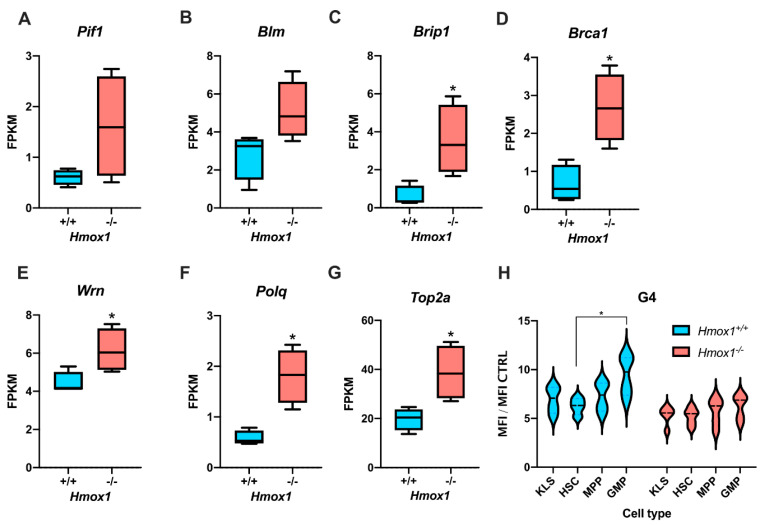
Mouse hematopoietic stem cells from *Hmox1^−/−^* mice had a higher expression of genes associated with G4 unwinding and showed lower G4 staining. The expression of (**A**) *Pif1*, (**B**) *Blm*, (**C**) *Brip1*, (**D**) *Brca1*, (**E**) *Wrn*, (**F**) *Polq*, and (**G**) *Top2a* was assessed with RNA-Seq in fluorescence-activated cell-sorting (FACS)-sorted phenotypic bone marrow hematopoietic stem cells (HSCs) (data source: [42]). The box-and-whiskers graphs show the median, 25th and 75th percentile (box) minimum, and maximum values (whiskers) of the FPKM (fragments per kilobase million), *n* = 4, * *p* < 0.05, Mann–Whitney test. (**H**) G4 staining shown as the mean fluorescence intensity (MFI) ratio of cells stained with anti-G4 antibodies and the background fluorescence (secondary antibodies only) in hematopoietic stem and progenitor cells (KLS), HSCs, multipotent progenitors (MPPs), or granulocyte-monocyte progenitors (GMPs) from *Hmox1^+/+^* and *Hmox1^−/−^* mice, *n* = 4–5, violin plot, * *p* < 0.05, two-way ANOVA with a Bonferroni post hoc test, for genotype *p =* 0.002.

In a previous study, we found that hematopoietic stem cells (HSCs) isolated from the bone marrow of young *Hmox1^−/−^* mice showed a transcriptional signature that was similar to that of aged wild-type HSCs [42]. We noticed that both young *Hmox1^−/−^* and old *Hmox1^+/+^* HSCs were characterized by a much higher expression of genes associated with the unwinding of G-quadruplexes (Figure 1A–G) and fewer G-quadruplexes (Figure 1H). Namely, *Hmox1^−/−^* HSCs had significantly higher levels of *Brip1* (Figure 1C), *Brca1* (Figure 1D)*, Wrn* (Figure 1E), *Polq* (Figure 1F), and *Top2a* (Figure 1G). We also showed the reduced quiescence and upregulated expression of genes associated with replication stress [42]. One could hypothesize that such a gene expression profile in *Hmox1^−/−^* HSCs may reflect the response of cells to increased G-quadruplex formation. The question is whether this putative protective response is effective. To check whether the induction of the G4-processing pathway is accompanied by changes in G4 levels, we isolated mouse bone marrow HSCs, multipotent progenitors (MPPs), granulocyte-monocyte progenitors (GMPs), and more heterogeneous fractions of hematopoietic stem and progenitor cells (KLS) from *Hmox1^+/+^* and *Hmox1^−/−^* mice and stained them with anti-G4 antibodies. The *Hmox1^−/−^* cells, regardless of their type, showed weaker staining (Figure 1H), suggesting that the upregulation of DNA helicases allowed for unwinding G-quadruplexes. On the other hand, in *Hmox1^+/+^* mice but not in *Hmox1^−/−^* mice, the staining for G4 was stronger in GMPs than in HSCs (Figure 1H).

### 3.2. The Effect of Hmox1 Deficiency on G-quadruplexes Was Cell-Type Specific

**Figure 2:**
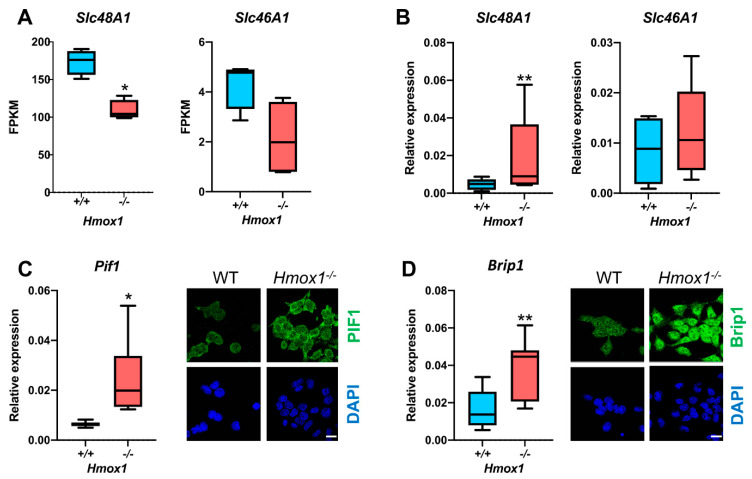
Expression of Slc48A1 and Slc46A1 in mouse *Hmox1^+/+^*or *Hmox1^−/−^* HSCs (**A**) and mouse *Hmox1^+/+^* or *Hmox1^−/−^* iPSCs (**B**). Expression of Pif1 (**C**) and Brip1 (**D**) at the mRNA (graphs) and protein (photos) levels in iPSCs. Gene expression was assessed with RNA-seq in FACS-sorted bone marrow HSCs [42] or with real-time RT-PCR in cultured iPSCs. The box-and-whiskers graphs show the median, 25th and 75th percentile (box) minimum, and maximum values (whiskers) of the FPKM (fragments per kilobase million) or relative expression in comparison to house-keeping genes (geometric mean for HPRT, B2M, and β-actin), *n* = 4, * *p* < 0.05, ** *p* < 0.01, Mann–Whitney test (HSCs) or Wilcoxon test (iPSCs). Representative pictures showing Pif1 and Brip1 immunostaining. Nuclei are labeled with DAPI. Scale bars: 20 µm.

Hematopoietic stem cells are quiescent and regulated by niche-derived signals that are Hmox1-dependent [42]. Therefore, it is difficult to distinguish between the direct and indirect effects of Hmox1 in HSCs. The expression of Hmox1 in HSC is very low, much lower than in cells forming the bone marrow niche [42]. Interestingly, HSCs have a very high expression of Slc48A1, the main heme transporter that regulates the intracellular heme availability through the endosomal or lysosomal compartment [43]. In *Hmox1^−/−^* HSCs, the Slc48A1 level was strongly reduced (Figure 2A). A similar tendency was visible for another heme importer, Slc46A1 (Figure 2A), while the genes coding for heme synthesis or heme export proteins were not affected (data not shown).

To check whether a decrease in G4 level is tightly associated with Hmox1 deficiency or can be affected by other genes of the heme metabolism pathway, we decided to repeat the analysis using different cell types. We generated mouse induced pluripotent stem cells (iPSCs) from *Hmox1^+/+^* and *Hmox1^−/−^* fibroblasts. The knockout of heme oxygenase-1 was confirmed with standard genotyping (Figure S1A). In these highly proliferating cells, in contrast to HSCs, Hmox1 deficiency is accompanied by the upregulation of Slc48A1 and an unchanged expression of Slc46A1 (Figure 2B). Similarly to HSCs, the expressions of G4-unwinding helicases (Pif1 and Brip1) were enhanced (Figure 2C,D).

**Figure 3:**
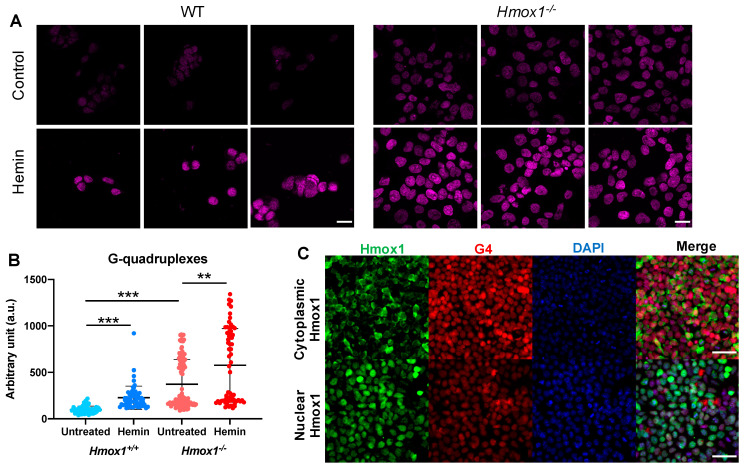
G-quadruplexes in iPSCs. (**A**) G4-specific staining (magenta) of *Hmox1^+/+^* or *Hmox1^−/−^* iPSCs that were untreated (control) or treated with hemin (2 μmol/L, 4 h) and (**B**) quantitative analysis of the fluorescence intensity. Analysis of cells from three independent experiments, Kruskall–Wallis test, ** *p* < 0.01, *** *p* < 0.001. Scale bars: 20 µm. (**C**) Staining for Hmox1 (green), G-quadruplexes (red), and DAPI (blue) in *Hmox1^−/−^* and *Hmox2^−/−^* iPSCs with re-introduced cytoplasmic or nuclear Hmox1. Scale bars: 50 µm.

The immunostaining of cultured cells showed that *Hmox1^−/−^* iPSCs had significantly higher levels of G-quadruplexes than their wild-type counterparts. Moreover, the G4 signal was clearly enhanced after the exposure of cells to hemin (2 µmol/L, 4 h) (Figure 3A). This indicates that Hmox1 did not regulate the stability of G-quadruplexes directly, but rather indirectly, possibly through the regulation of heme availability. Hemin did not affect the expression of Pif1 and Brip1 helicases (Figure S2). Quantitative analysis also revealed that Hmox1-deficient cells were not homogenous. Instead, there were cells with relatively low levels of G-quadruplexes and fractions with strong G4 signals (Figure 3B). Such a highly G4-stained fraction was absent from the *Hmox1^+/+^* iPSCs. This suggests that in some physiological states (e.g., during DNA replication), the disturbance in heme degradation resulting from Hmox1 deficiency impaired G4 unwinding.

Heme oxygenase-1 can translocate to the nucleus, especially in response to treatment with hemin ([44,45], our unpublished observations). To check whether cellular localization can modulate the influence of Hmox1 at the G4 level, and to avoid the potentially interfering effect of Hmox2, we knocked out the Hmox2 gene from the *Hmox1^−/−^* iPSCs using Crispr/Cas9, as evidenced using Western blotting and with immunostaining (Figure S1A–C). Then, using lentiviral vectors, we introduced Hmox1 with a nuclear localization signal (NLS) or with a nuclear export signal (NES) to iPSCs lacking both endogenous Hmox1 and Hmox2. Such cells were then stained for both Hmox1 and G-quadruplexes. Interestingly, cells with predominantly nuclear Hmox1 stained weaker for G-quadruplexes than cells with cytoplasmic Hmox1 (Figure 3C). Thus, this experimental model indicated that the nuclear translocation of Hmox1 may affect G4 stability.

### 3.3. HMOX1 Localized Close to G-quadruplexes in DNA

To determine whether HMOX1 can be localized in the proximity of G-quadruplexes, we decided to use a proximity ligation assay (PLA). Because this assay has so far not been widely used for the detection of co-localization between proteins and G4 before, we had to optimize the method. Experiments were performed in HEK293T cells, which are fast-growing and easily transfected.

The same as for a conventional PLA, in our application, the target molecules were labeled with specific antibodies. Primary antibodies are crucial for both the selectivity and sensitivity of detection; thus, the first step in our study was to validate them. To date, two different antibodies recognizing G4 structures were developed and both were successfully used to visualize G-quadruplex structures in human cells. A 1H6 clone was produced via the immunization of mice with stable G4-DNA [18] and a BG4 clone was made using phage display with a library of different single-chain antibody clones [17]. The G4 structures labeled using the immunofluorescence technique are seen as nuclear foci that are sensitive to the treatment with DNase [17,18].

**Figure 4:**
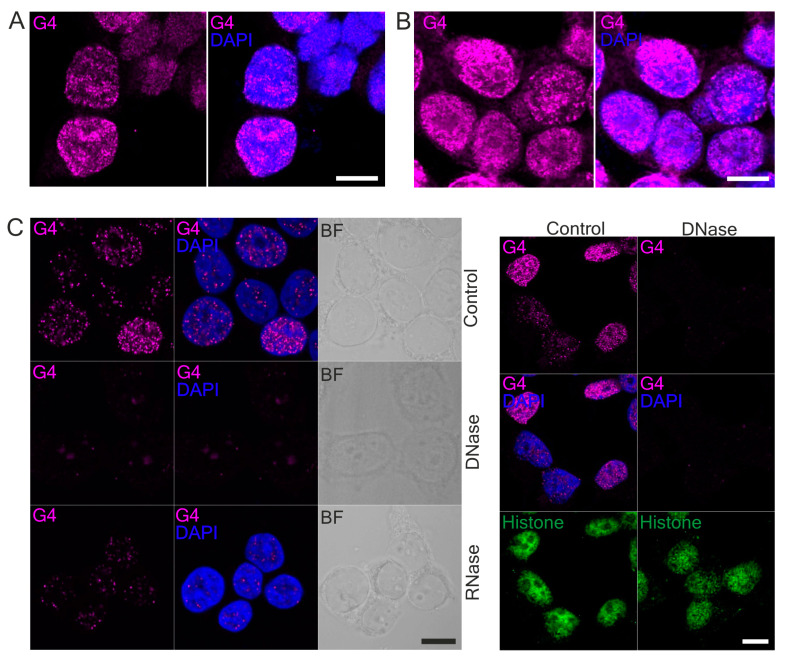
G4 immunolabelling in HEK293T cells. (**A**) Mouse and (**B**) goat 1H6 antibodies were used to visualize the G4 structures (magenta). (**C**) G4-specific staining (mouse antibodies) disappeared after the treatment with DNase but were still visible after the treatment with RNase. Cells were fixed in methanol. Nuclei were counterstained with DAPI (blue) or histone H3 (green), where transmission bright-field (BF) images show the cell morphologies. Scale bars: 10 μm.

Using two different commercially available 1H6 antibodies, we found similar staining patterns in HEK293T cells (Figure 4) to those previously reported by Henderson et al. who tested 1H6 antibodies in a HeLa cell line [18]. Strong, granular nuclear staining was observed in cells stained using either mouse (Figure 4A) or goat (Figure 4B) antibodies against G4. However, goat antibodies also exhibited a strong signal in the cytoplasm. Importantly, the immunofluorescence foci in the nuclei almost completely disappeared after DNase treatment but much less after RNase treatment (Figure 4C). We also noticed that the fluorescence signal was stronger when the cells were fixed in methanol in comparison to when the cells were fixed in PFA (Figure S3). Finally, negative controls in which primary antibodies were omitted confirmed the specificity of the reaction (Figure S4). Altogether, our protocol utilizing 1H6 antibody allowed for the detection of G-quadruplexes, primarily DNA–G4 structures.

For a better understanding of the biological functions of G4, it is crucial to identify and characterize proteins that interact with G-quadruplexes. As a PLA positive control, we decided to visualize known protein–protein partners. We chose two nuclear proteins KU70 and KU80, which heterodimerize and form complexes involved in the main DNA double-strand break repair pathway in mammals [46]. Next, to verify that PLA was indeed suitable for a direct *in cellulo* detection of G4–protein partners, we used this method to visualize G4 interactions with a previously identified endogenous partner, namely, BRIP1 protein. BRIP1 is a DNA helicase involved in DNA–G4 resolving [47]. Finally, we attempted to investigate whether HMOX1 is a potential G4 binding partner since our RNA-seq data showed the strong upregulation of genes responsible for resolving G-quadruplexes in *Hmox1*-deficient cells.

**Figure 5:**
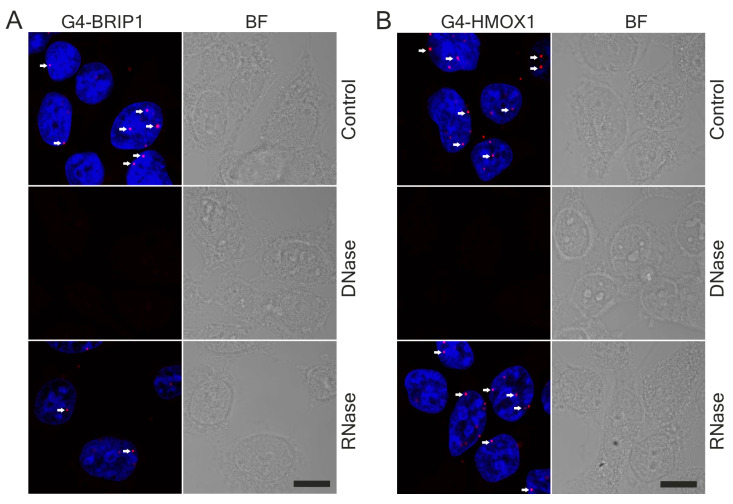
In situ proximity ligation assay (PLA) in HEK293T cells. (**A**) G4-BRIP1 and (**B**) G4-HMOX1 interactions (red dots) were visualized in the control group and after DNase or RNase digestion. Most of the G4 interactions were localized in the nucleus (arrows), but some were also present in the cytoplasm. Cells that were fixed in methanol and mouse 1H6 antibodies were used. Nuclei were counterstained with DAPI (blue), where the transmission bright-field images (BF) show the cell morphologies. Scale bar: 10 μm.

Our results showed that we were able to detect nuclear signals for both G4-BRIP1 (Figure 5A) and G4-HMOX1 (Figure 5B) interactions using PLA. These interaction spots were localized in the nucleus, but we also noticed a small proportion of them in the cytoplasm. The positive control (detection of KU70 and KU80 dimers) showed strong staining and the highest number of nuclear foci (Figure S5). Consistent with immunofluorescence experiments, the DNase treatment completely abolished the G4-BRIP1 and G4-HMOX1 PLA signal (Figure 5A,B, respectively), but resulted in only a partial decrease of the KU70-KU80 signal (Figure S5). In turn, the RNase treatment did not cause an appreciable decrease in the PLA signal for the investigated interactions (Figure 5A–C and Figure S5), which suggests that we detected interactions with DNA–G4 structures. Finally, controls without primary antibodies (Figure S6A) or with only one primary antibody (Figure S6B) confirmed the specificity of the reactions.

It is noteworthy that standard immunolabeling showed that DNA digestion abolished G4 staining (Figure 4C), while the BRIP1 and HMOX1 proteins in the nucleus remained intact (Figure S7). This confirmed that the lack of in situ PLA signal after the DNA digestion (Figure 5) resulted specifically from destroying G4 structures localized in the DNA.

### 3.4. G4-HMOX1 Interaction Could Be Detected Using Flow Cytometry PLA

**Figure 6:**
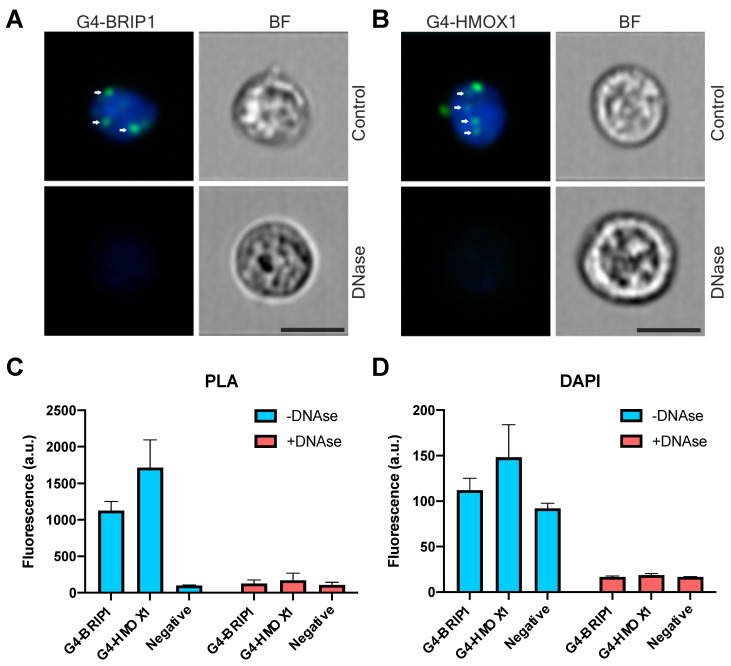
Detection and quantification of the PLA signal at a single-cell level in HEK293T cells using flow cytometry. (**A**) G4-BRIP1 and (**B**) G4-HMOX1 interactions (green dots) were visualized in the control group and after DNase digestion. Most of the G4 interactions were localized in the nucleus (arrows) but some also appeared in the cytoplasm. Nuclei were counterstained with DAPI (blue), where the transmission bright-field images (BF) show the cell morphologies. Cells were fixed in methanol and mouse 1H6 antibodies were used. Scale bars: 10 μm. (**C**) Mean fluorescence of G4-BRIP1 and G4-HMOX1 PLA signal and (**D**) DAPI in the control cells and after DNase digestion. Negative controls were cells stained with secondary antibodies only. Data is shown as mean + SEM (standard error of the mean).

To further explore whether PLA can also be used for quantitative analysis, we studied the proximity of G4 with proteins of interest using flow cytometry. First, we visualized the PLA signal in single cells using Image Stream (Figure 6A,B). PLA-specific dots were seen in the nucleus but also in the cytoplasm. Similarly, to the in situ data, PLA foci disappeared after DNase digestion (Figure 6A,B). Analysis of the fluorescence intensity (Figure 6C) showed that the G4–HMOX1 signal was slightly higher than for G4–BRIP1 and that after the DNase treatment, the PLA signal was at the same level as for the negative controls. Analysis of the DAPI fluorescence showed the efficiency of DNase digestion (Figure 6D). Figure S8 demonstrates the negative controls.

Collectively, the data show that PLA could be used for quantitative analysis of G4 interactions in cells using flow cytometry.

### 3.5. G-quadruplex and Hmox1 Interaction Could Be Detected in Primary Mouse Hematopoietic Stem Cells

**Figure 7:**
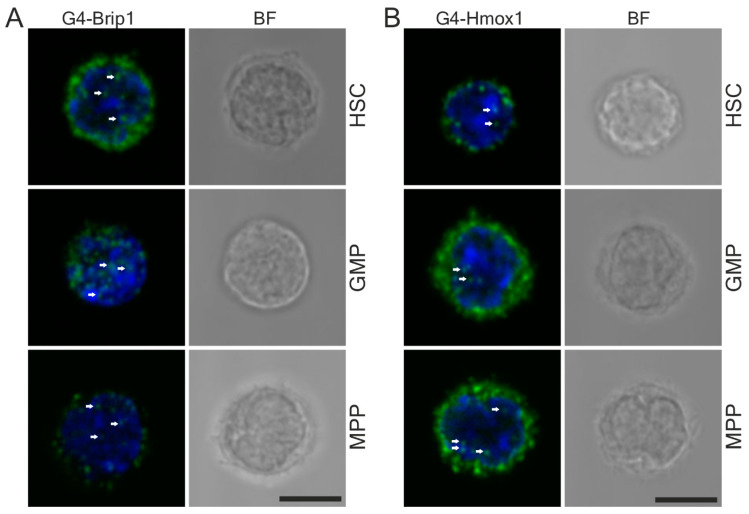
Detection of the PLA signal in sorted hematopoietic stem cells. (**A**) G4–Brip1 and (**B**) G4–Hmox1 interactions (green dots) were visualized in HSCs and GMP and MPP cells. G4 interactions were visible in the nucleus (arrows) and in the cytoplasm. Nuclei were counterstained with DAPI (blue), where the transmission bright-field images (BF) show the cell morphologies. Cells were fixed in PFA and goat 1H6 antibodies were used. Scale bars: 5 μm.

To test whether we could detect G4 partners in rare populations of cells using PLA, we combined this technique with FACS. Indeed, by applying our protocol, we were able to demonstrate G4–Brip1 and G4–Hmox1 interactions in primary mouse hematopoietic stem cells and progenitor cells (Figure 7A,B, respectively). The G4–Hmox1 signal was seen in the nucleus, but was also abundant in the cytoplasm, especially in the progenitor cells (MPP and GMP). Figure S9 demonstrates the negative controls.

Together, our data indicate that the PLA technique using 1H6 antibodies allowed for specific visualization of G4 protein partners in cells. Importantly, we revealed that the HMOX1 protein closely co-localized with G-quadruplexes formed by nucleic acids. It seems, however, that despite this proximity, the HMOX1 protein did not bind to DNA–G4 directly, since we were unable to detect any signal using a standard chromatin immunoprecipitation assay (Figure S10). This observation is consistent with the supposition that the effect of HMOX1 on G-quadruplexes may result mainly from the regulation of local heme availability.

### 3.6. Potential Interaction between Heme and G-quadruplexes Did Not Affect the Hmox1 Expression

Additionally, we examined whether the increased level of G-quadruplexes can act as a buffer to sequester free heme in living cells. Such an ability was recently demonstrated in HT1080 fibrosarcoma [48]. We used HEK293T cells to check how the transfection with oligonucleotides, which form G-quadruplexes, affected the expression of Hmox1 and levels of free heme. To do so, we transfected HEK293T cells with G4-forming ckit87up or control dsDNA oligonucleotides. The effectiveness of the transfection was confirmed by staining the cells with N-methylmesoporphiryne IX (NMM, 10 μmol/L), which increased its fluorescence in the presence of G-quadruplexes. Indeed, HEK293T cells showed higher NMM fluorescence when transfected with 18 nM ckit87up but not with control DNA (Figure S11A). Then, cells were treated with different concentrations of hemin (2–20 µM/L, 3h). Transfection with heme-sensitive reporter plasmids allowed for evaluating changes in free heme availability. An increase in the free heme level was visible in the cytoplasm and nucleus of cells treated with the highest concentration of hemin, but we were not able to detect any differences between cells transfected with G4-forming and control oligonucleotides (Figure S11B). Similarly, the expression of *Hmox1* was dose-dependently induced by hemin, regardless of the G4 transfection (Figure S11C).

## 4. Discussion

A growing body of evidence connects G-quadruplexes with critical biological functions, like DNA replication, gene expression regulation, or translation. In recent years, G4 structures have been extensively characterized in vitro using different spectroscopic measurements. While those experiments were mostly based on chemically synthesized sequences folded into various G4 conformations, G-quadruplexes’ formation and function in mammalian cells remain poorly understood. Therefore, much of the recent efforts should aim toward developing tools for detecting and monitoring G-quadruplex structures in living cells. To date, small-molecule G4 binders have been successfully applied to examine cellular G-quadruplexes [15,49]. On the other hand, antibodies have also attracted significant attention from researchers for the direct visualization of G4 in cellulo [17,18]. Here, we used G4-specific antibodies to visualized G4 structures in human and mouse cells. Our immunostaining data are consistent with those published by Henderson et al. and confirmed that 1H6 antibodies recognize primarily DNA-G4 primarily [18].

The supposed roles of G-quadruplexes in living cells have also stimulated the search for their protein partners. During DNA replication, G-quadruplex structures need to be actively resolved. Indeed, numerous DNA helicases, such as BLM, BRIP1, PIF1, or WRN, have the ability to unwind G4 structures (for a review, see reference [19]). However, such interactions were validated mainly under non-physiological conditions. A few attempts have been made to confirm the similar role of helicases in living cells. Using yeasts, Paeschke et al. indicated that Pif1 suppresses both G-quadruplex-associated DNA damage and telomere lengthening, while Ribeyre et al. reported that the absence of Pif1 promotes the genetic instability of G4 [20,21]. In turn, experiments on human cells showed that the lack of WRN and BLM dysregulates global transcription by targeting DNA-G4 [50]. Finally, Castillo Bosch et al. developed a model system using *Xenopus* egg extracts and single-stranded DNA templates, and evidenced that Brip1 unwinds G4 structures and promotes DNA synthesis [22].

Besides helicases, a wide range of G4 binding proteins has been recognized so far. One of the first identified G4 partners was nucleolin, which has an affinity for both DNA–G4 and RNA–G4 sequences [51]. Here, using the PLA technique, we directly showed that HMOX1 localized in the proximity of G-quadruplexes and the *Hmox1* expression level may modulate the accumulation of G4.

We focused on HMOX1 for two reasons. First, our RNA-seq data [42] showed that hematopoietic stem cells from *Hmox1^−/−^* mice have a higher expression of genes associated with the unwinding of G-quadruplexes. Second, numerous in vitro experiments evidenced that G-quadruplexes tightly bind heme, which stabilizes them and impairs their unwinding by helicases [28,52,53,54]. More recently, Gray et al. confirmed that in human cells, G-quadruplexes sequester free heme, thus affecting genomic stability [48]. HMOX1 is a key enzyme for removing excess heme from different cellular compartments [55]. Therefore, we suppose that HMOX1 might directly facilitate the destabilization and resolving of G4 structures through the degradation of heme.

We compared the effect of Hmox1 deficiency on G-quadruplex abundance in two cell types: hematopoietic stem cells from *Hmox1^−/−^* mice and iPS cells generated from fibroblasts of *Hmox1^−/−^* mice. In both models, *Hmox1^−/−^* cells had an elevated expression of G4-unwinding helicases, namely *Brip1* and *Pif1*. The mechanism underlying this elevation is unclear. We believe that it is not directly related to the *Hmox1* status, as treatment of *Hmox1^+/+^* iPSCs with hemin did not affect the *Pif1* and *Brip1* levels. *Brip1* is a direct target of the E2F2 transcription factor [56]. We found [42] that several direct targets of E2F2 are upregulated in *Hmox1^−/−^* HSCs, which is accompanied by the deregulation of the G1/S cell-cycle checkpoint and reduced cell quiescence.

We assumed that if Hmox1 was the key factor regulating the heme availability, and thus influencing G4 stability, we would observe a significant increase in cellular G4 levels in both Hmox1-deficient cellular models. Indeed, in iPS cells, the *Hmox1*-deficiency was associated with more abundant G-quadruplexes, despite an increased expression of *Pif1* and *Brip1*. Moreover, the treatment of cells with exogenous heme led to a further increase in G4. This observation supports the potential role of HMOX1 in the regulation of G4 stability, which is possibly mediated by free heme removal.

However, in hematopoietic stem cells, we found the opposite relation. *Hmox1^−/−^* HSCs had fewer G-quadruplexes than their *Hmox1^+/+^* counterparts, indicating that G4-unwinding helicases were effective. Interestingly, the upregulation of G4-helicase expressions was accompanied by a significant decrease in the expression of *Msh2* and *Msh6* ([42], data not shown) coding for proteins (MutS homolog 2 and 6) that bind to G4 and inhibit Brip1 activity [47]. This might additionally improve G4-unwinding. We suppose, however, that the most important are the tissue-specific mechanisms regulating heme levels. HSCs have very high expression levels of the *Slc48A1* transporter, which is involved in the import of heme from endosomes to the cytosol. This expression is significantly reduced in *Hmox1^−/−^* HSCs, which may result in the decreased uptake of heme. Recently, Canesin et al. described heme-overload-induced changes in expressions of cell-cycle regulating genes [11]. We checked that these changes are not reflected by the gene expression profile in *Hmox1^−/−^* HSCs ([42], data not shown), suggesting that *Hmox1^−/−^* HSCs do not have significantly elevated free heme. Importantly, the expression of *Hmox1* in murine HSCs is very low, much lower than that in cells of bone marrow niche. HSCs seem to rely more on the enzymatic activity of Hmox2, whose expression is high and comparable to that in the niche cells [42]. Taken together, the comparison of two cellular models shows that Hmox1 can affect G4 accumulation but it is not a universal player, and its influence can be masked by other factors.

Using the PLA technique, we tried to show a direct interaction between G4 structures and BRIP1 protein in cells as a kind of positive control. There are strong indirect premises supporting such an interplay. As such, it is known that cells derived from BRIP1-depleted patients accumulate deletions in the vicinity of G4 genomic sequences [47] and have elevated levels of DNA damage and apoptosis upon exposure to the G4-interactive compound telomestatin [57]. Moreover, a chicken *Brip1* knockout cell line was shown to have more G-quadruplexes detected using an immunostaining reaction. We demonstrated for the first time that BRIP1 protein is in close proximity with G4 in the cell nucleus of HEK293T cells. A similar approach was used by Stroik et al., who used a proximity ligation assay to demonstrate in vivo that RTEL1 helicase is a G4 partner [58]. Finally, direct colocalization of G4 with PIF1 helicase in U2OS cells was shown using fluorescently labeled pyridostatin, a small G-quadruplex-binding molecule [15]. In contrast to this experiment, in which GFP-hPif1α construct was overexpressed, PLA allowed for visualization of G4 interactions in intact cells. Thus, this approach can provide evidence for molecular interactions that are naturally occurring in unperturbed cells.

The results of PLA could provide an understanding of the enigmatic role of HMOX1 in the cell nucleus. Using iPS cells lacking both endogenous heme oxygenases but expressing transgenes coding for nuclear or cytoplasmic form of Hmox1, we showed that the nuclear localization may promote G4 removal. One possibility is that the nuclear Hmox1 removes excess heme locally, thereby more effectively destabilizes G4. However, the enzymatic activity of Hmox1 in the nucleus is still the matter of debate [59]. The second possibility is that Hmox1 is a part of the G4-resolving complex or facilitates the formation of such a complex.

It is worth noticing that we were unable to detect a direct Hmox1-DNA interaction using the routine chromatin-immunoprecipitation assay. However, co-immunoprecipitation coupled to mass spectrometry [60] has demonstrated that HMOX1 can directly interact with other proteins. Intriguingly, we noticed that 12 out of 15 recognized partners of HMOX1 are known to play a role in the processing of G-quadruplexes. There are proteins involved in both DNA replication and repair (PARP1, XRCC6, RAD51C, TOP1, SFPQ, NONO, BMH1) and RNA synthesis, splicing, and translation (hnRNPA1, hnRNPK, hnRNPL, DHX9, NCL). It will be important to compare the composition and activity of the G4-resolving complex in the presence and absence of HMOX1.

It seems that HMOX1 is involved in the removal of heme released from G-quadruplexes. This supposition is supported by the results of Gray et al. who showed that the displacement of G4-bound heme brought about by PhenDC3 (known G4 ligand) administration leads to a 30-fold induction of *HMOX1* expression as a result of the rapid elevation of free heme [48]. This observation is also in line with a recent report that G4 can act as a heme-harvesting buffer [61]. We transfected HEK293T cells with oligonucleotides, which form G-quadruplexes, to see if it changed the levels of free heme or *HMOX1* expression, but in such an experimental setting, there were no differences. We suppose that the putative effects of exogenous G4-forming oligonucleotides may be masked by other cellular buffering systems, primarily proteins, such as GAPDH [62]. Thus, the physiological significance of G4 as heme sequesters in different cell types is still an open question. Both G4-BRIP1 and G4-HMOX1 interactions were detected by PLA as bright foci located in the cell nucleus, which were sensitive to DNase treatment. However, we also observed sporadic spots in the cytoplasm. Since the 1H6 antibody has a higher affinity for DNA-G4 than for RNA-G4 [18] and that the PLA signal in the cytoplasm disappeared after DNase digestion, we suspect that these dots might correspond to the G4 in mitochondrial DNA. Our results support the findings of Hoffmann et al., who demonstrated, using 1H6 antibodies and immuno-electron microscopy, the presence of single immuno-gold particles in the cytoplasmic site of the endoplasmic reticulum and in mitochondria [63]. We also noticed that G4-specific staining in the nucleus was weaker after RNase treatment. Hence, we suppose that the antibodies can also bind to some G4 on RNA. Indeed, a computational approach has been carried out to locate the RNA G-quadruplex in the 5′ and 3′ untranslated regions (UTRs) of mRNA. Moreover, in vitro and in vivo experiments have indicated that G4 in mRNA may play key roles in pre-mRNA processing (including splicing and polyadenylation) [64]. Although some unspecificity of enzymatic treatment cannot be excluded, we suppose that RNase digestion mainly removed mRNA and the G4 located on it.

The measurement of fluorescence using flow cytometry and the quantification of in situ PLA dots in the nuclei of HEK293T cells (data not shown) suggests more G4–HMOX1 interactions in comparison with G4–BRIP1. Such a comparison should be interpreted very carefully, as it can just be a result of different antibody affinities; nevertheless, it supports the conclusion that interplay between HMOX1 and G4 is not a marginal event. This possibly more abundant signal from HMOX1 can also be explained by the fact that various G-quadruplexes in the cell are probably recognized by different proteins. For example, Castillo Bosch et al. revealed that some G-quadruplex structures might be resolved in a BRIP1-independent manner [22]. Finally, as shown by Biffi et al., there is a clear relationship between the cell cycle and G-quadruplex formation, with the highest number of G4 foci during the S phase and the lowest at G0/G1 [17]. Indeed, we observed fractions of cells with low and high G4 levels in the same sample, which probably depended on the cell cycle stage since we did not synchronize the cell culture. It also has to be mentioned that as in situ PLA is a method based upon equilibrium reactions and several enzymatic steps, only a fraction of the interacting molecules was detected [35].

Finally, standard PLA protocol based on confocal microscopy analysis does not allow for quantification linked to a specific cell population. We tried to overcome this limitation via a combination of fluorescence-activated cell sorting and in situ PLA. This approach allowed for quantitative analysis of G4–protein interactions in various and even rare cell populations. Such experiments are technically challenging because they require additional staining with multiple surface markers for the identification of specific cells. Recently, Avin et al. used a similar approach to quantify protein–protein interactions and post-translational modifications in rare immune populations [65]. They coupled the PLA assay with a conventional immunofluorescence technique, which allowed for multiparametric fluorescent and morphological analysis. Unlike their approach, where the PLA signal was detected using flow cytometry, we analyzed sorted cells using confocal microscopy. Both strategies have their weak and strong points. The use of flow cytometry allows for the characterization of thousands of cellular events, along with statistical analysis. It is also the fastest and least technically challenging method since the procedure on the slide is omitted. On the other hand, the resolution of confocal microscope scans is far better than a cytometer; therefore, it is easier to follow the intracellular localization of studied interactions. Moreover, it has been shown that the flow-cytometer-based technique generates some portion of false-positive PLA-labelled cells [65], which we also noticed in our experiments (data not shown). Thus, additional filtering of the signal proposed by Avin et al. might be needed for precise and correct quantification of the data [65]. Taken together, cell sorting followed by the PLA seems to be a suitable procedure for the spatial analysis of G4–protein interactions in the rare cell population of choice and can be used universally in different biological systems. The major limiting factor is the availability of a flow-cytometry-grade antibody to be used as a marker.

In summary, we found that HMOX1 colocalizes with G-quadruplexes, similar to G4-unwinding helicases. HMOX1 influences G4 accumulation, but rather as one of the proteins regulating heme availability, not as a rate-limiting factor. It is noteworthy that we demonstrated that PLA offers a powerful tool for the qualitative and quantitative analysis of G4–protein interactions at a single-cell level.

## Data Availability

All data in the manuscript is available from the corresponding author upon the request.

## Supplementary Materials

The following are available online at https://www.mdpi.com/2076-3921/10/1/94/s1. Figure S1. (**A**) Genotyping of wild type, *Hmox1^−/−^* iPS (iPSC HO1 KO), *Hmox1^−/−^Hmox2^−/−^* iPS cells (iPS HO1 KO HO2 KO), gDNA from *Hmox1*^+/+^ and *Hmox1*^−/−^ mice was used as a control, (**B**) Western blotting for Hmox1 and Hmox2 in wild type, *Hmox1*^−/−^ iPS (iPS HO1 KO), *Hmox1^−/−^Hmox2^−/−^* iPS cells (iPS HO1 KO HO2 KO), (**C**) Hmox1 and Hmox2 ICC staining in wild type, *Hmox1*^−/−^ iPS (iPS HO1 KO), *Hmox1^−/−^Hmox2^−/−^* iPS cells (iPS HO1 KO HO2 KO); scale bar: 20 mm (**D**) localization of Hmox1 in iPS cell lines transduced with lentiviral vectors. iPS-Hmox1-NES showed median similarity ratio between nuclei and Hmox1 staining 0.86 while iPS-Hmox1-NLS 1.89. Moreover, 44.7% iPS-Hmox1-NES, and 76.4% iPS-Hmox1-NLS cells had similarity ratio higher than 1. Figure S2. RealTime PCR analysis. Expression of *Pif1* and *Brip1* genes in *Hmox1*^+/+^ and *Hmox1*^−/−^ iPSC after 2 μmol/L hemin treatment (24h incubation). Box and whiskers graphs show median, min and max values of relative expression in comparison to house-keeping genes (geometric mean for HPRT, B2M and β-actin), *n* = 3. Figure S3. G4 immunolabelling on HEK293T cells fixed in PFA. (**A**) Mouse and (**B**) goat 1H6 antibodies were used to visualize G4 structures (magenta). Top panel represent G4-specific staining while bottom panels show negative controls in which only secondary antibodies were used. Nuclei were counterstained with DAPI (blue). Scale bar: 10 µm. Figure S4. Negative controls for G4 immunolabelling on HEK293T cells fixed in methanol. Images represent negative controls for the pictures showed in (**A**) Figure 1A, (**B**) Figure 1B, (**C**) Figure 1C. Nuclei were counterstained with DAPI (blue) and transmission bright-field (BF) images are showing the cell morphology. Scale bar: 10 µm. Figure S5. Positive control for in situ PLA on HEK293T cells. KU70 and KU80 interactions (red dots) are visualized in the control group and after DNase or RNase digestion. Most of the interactions were localized in the nucleus but some also presented in the cytoplasm. Cells were fixed in methanol. Nuclei were counterstained with DAPI (blue) and transmission bright-field images (BF) showing the cell morphology. Scale bar: 10 µm. Figure S6. Negative controls for in situ PLA on HEK293T cells. (**A**) Controls with no primary antibodies used for control group and for cells after DNA or RNA digestion. (**B**) Controls with only one primary antibody used for PLA. Cells were fixed in methanol. Cell nuclei were counterstained with DAPI (blue channel) and transmission bright-field images (BF) showing the cell morphology. Scale bar: 10 µm. Figure S7. Immunolabelling of BRIP1 and HMOX1 proteins on HEK293T cells. (**A**) BRIP1 and (**B**) HMOX1 were stained (magenta) in control group and after DNA digestion. Cells were fixed in methanol and nuclei were counterstained with DAPI (blue). Scale bar: 10 µm. Figure S8. Detection and quantification of PLA signal at a single-cell level in HEK293T cells using flow cytometry–negative controls. Scale bar: 5 µm. Figure S9. Detection of PLA signal in sorted hematopoietic stem cells–negative controls. Scale bar: 5 µm. Figure S10. HMOX1 does not bind directly to DNA. (**A**) Input step of ChIP protocol; chromatin isolated from iPSC *Hmox1*^+/+^ treated (or not) with 2 µmol/L hemin and incubate for 4 h. *n* = 3 (**B**) Agarose gel after immunoprecipitation, reversal of cross-links and DNA purification. Figure S11. G-quadruplex oligonucleotides do not affect Hmox1 expression and free heme levels in HEK293T cells. (**A**) HEK293 cells transfected with 18 nmol/L ckit87up oligonucleotides show enhanced N-methylmesoporphyrin IX fluorescence, *N* = 3, * *p* < 0.05, One-Way ANOVA with Bonferroni post hoc test (**B**) Ratio of green (eGFP) to red (mKATE2) fluorescence of cytoplasmic and nuclear heme sensors in HEK293T cells transfected with G4-forming oligonucleotides or control DNA and treated with hemin. Green fluorescence of eGFP is quenched by heme. *N* = 3, bars show average ratio + standard deviation (**C**) Transfection with ckit87up oligonucleotides does not affect Hmox1 expression in HEK293 cells, *n* = 4, Two-Way ANOVA.
