# Supplementary material for: Proximity Ligation Assay Detection of Protein–DNA Interactions—Is There a Link between Heme Oxygenase-1 and G-quadruplexes?
Source: Antioxidants (Basel). 2021 Jan 12;10(1):94. doi: 10.3390/antiox10010094 (PMC7827836; doi:10.3390/antiox10010094)
Supplement: Supplementary file 1 [file antioxidants-10-00094-s001.pdf]

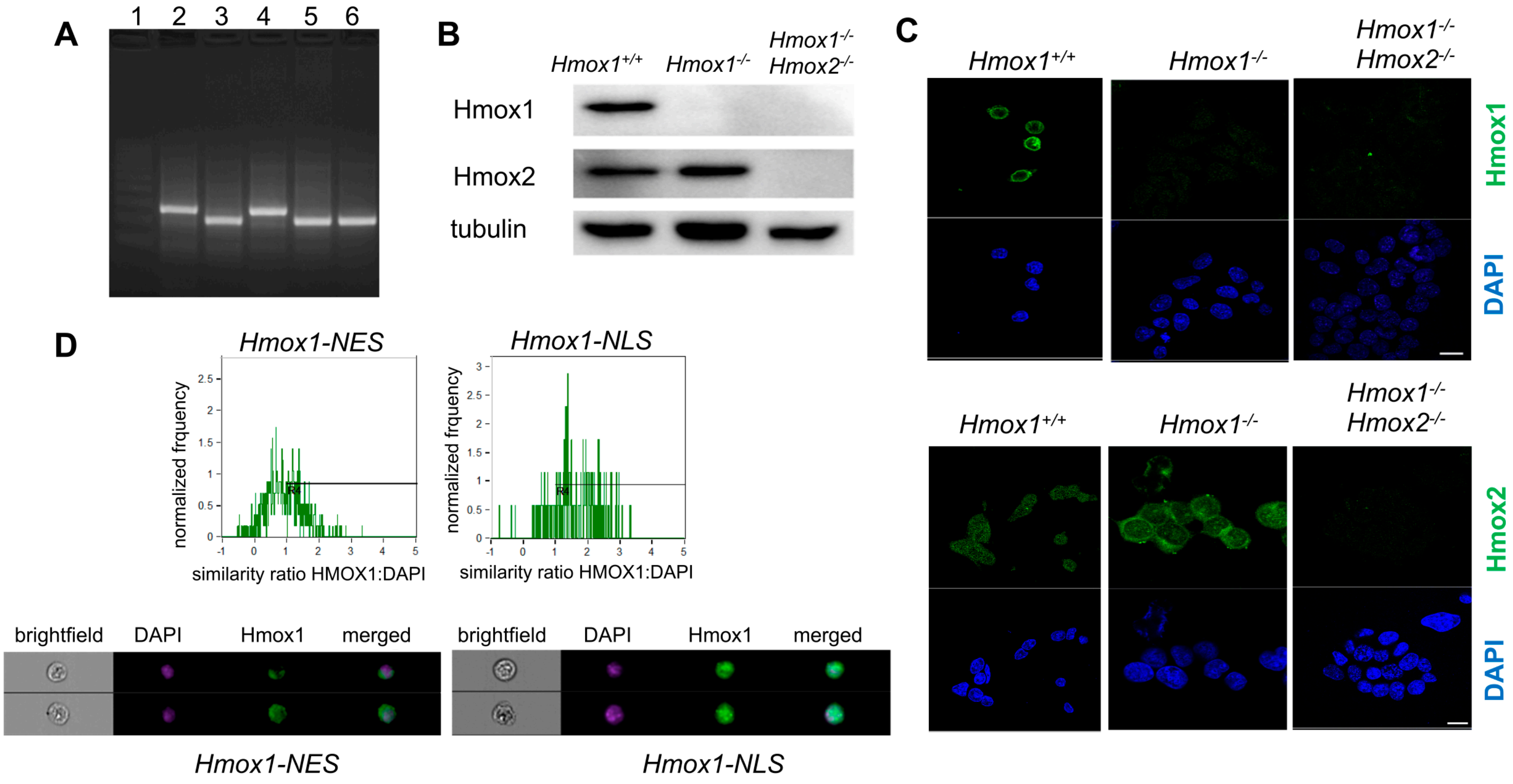

Figure S1. A) Genotyping of wild type, *Hmox1*$^{-/-}$ iPS (iPSC HO1 KO), *Hmox1*$^{-/-}$*Hmox2*$^{-/-}$ iPS cells (iPS HO1 KO HO2 KO), gDNA from *Hmox1*$^{+/+}$ and *Hmox1*$^{-/-}$ mice was used as a control, (**B**) Western blotting for Hmox1 and Hmox2 in wild type, *Hmox1*$^{-/-}$ iPS (iPS HO1 KO), *Hmox1*$^{-/-}$*Hmox2*$^{-/-}$ iPS cells (iPS HO1 KO HO2 KO), **(C)** Hmox1 and Hmox2 ICC staining in wild type, *Hmox1*$^{-/-}$ iPS (iPS HO1 KO), *Hmox1*$^{-/-}$*Hmox2*$^{-/-}$ iPS cells (iPS HO1 KO HO2 KO); scale bar: 20 µm **(D)** localization of Hmox1 in iPS cell lines transduced with lentiviral

vectors. iPS-Hmox1-NES showed median similarity ratio between nuclei and Hmox1 staining 0.86 while iPS-Hmox1-NLS 1.89. Moreover, 44.7% iPS-Hmox1-NES, and 76.4% iPS-Hmox1-NLS cells had similarity ratio higher than 1.

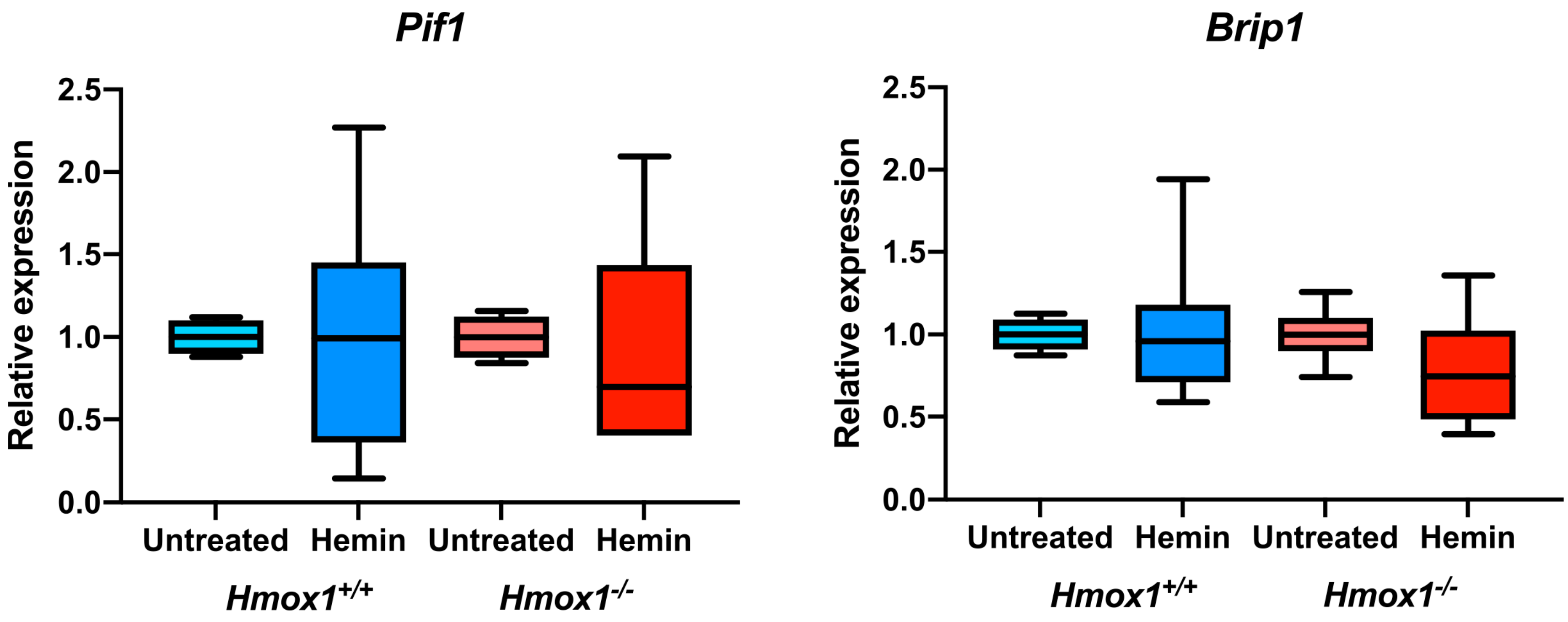

Figure S2. RealTime PCR analysis. Expression of *Pif1* and *Brip1* genes in *Hmox1*$^{+/+}$ and *Hmox1*$^{-/-}$ iPSC after 2µM hemin treatment (24h incubation). Box and whiskers graphs show median, min and max values of relative expression in comparison to house-keeping genes (geometric mean for HPRT, B2M and β-actin), n=3.

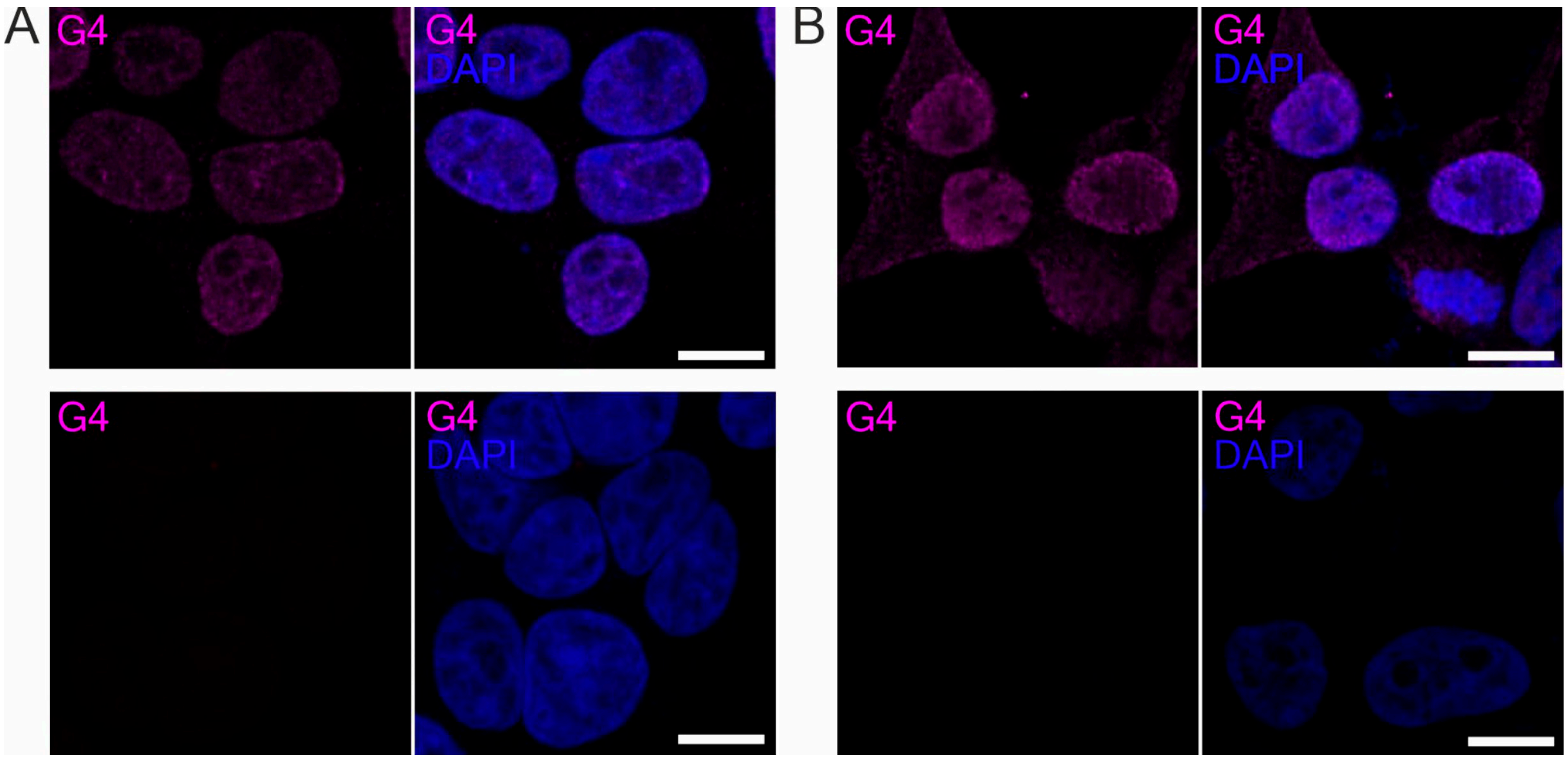

Figure S3. G4 immunolabelling on HEK293T cells fixed in PFA. (A) Mouse and **(B)** goat 1H6 antibodies were used to visualise G4 structures (magenta). Top panel represent G4-specific staining while bottom panels show negative controls in which only secondary antibodies were used. Nuclei were counterstained with DAPI (blue). Scale bar: 10 µm.

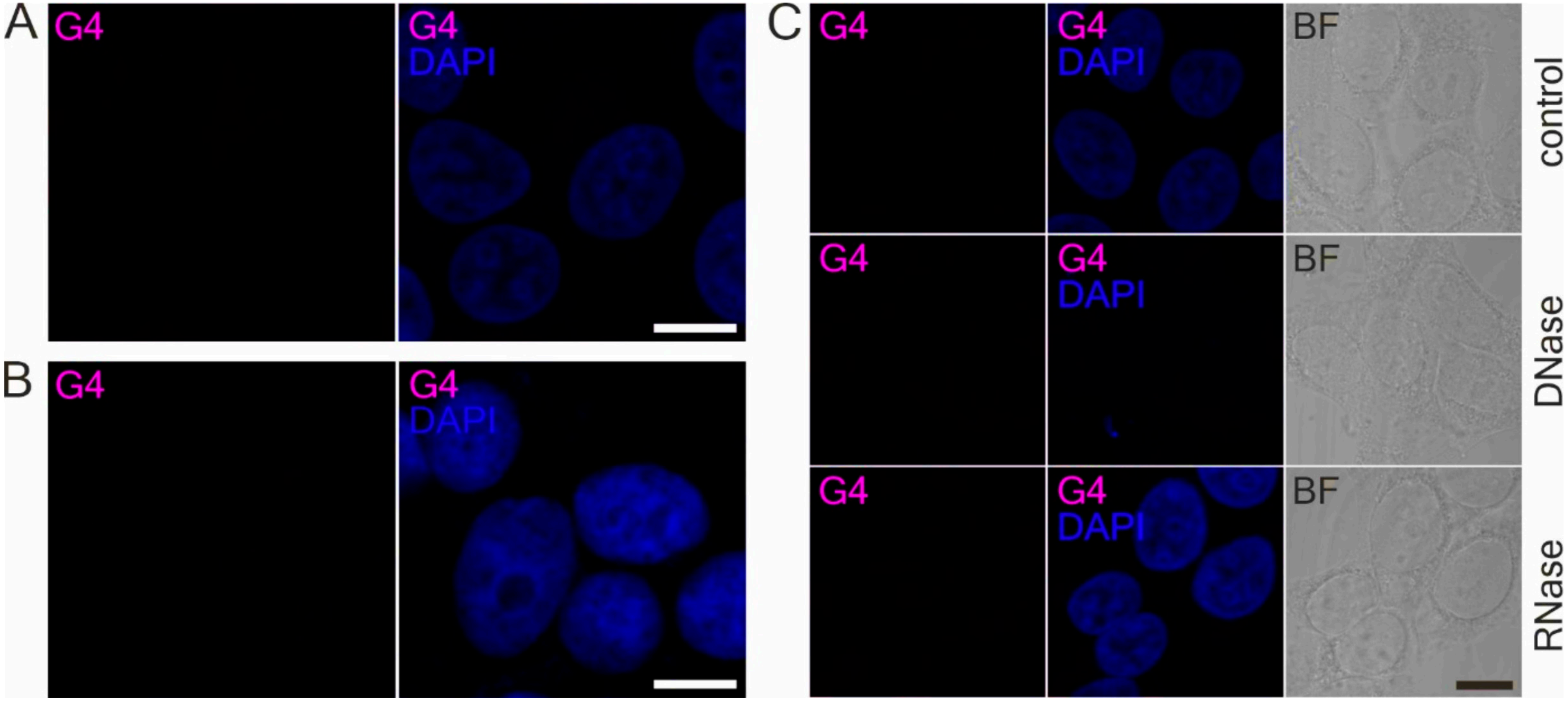

Figure S4. Negative controls for G4 immunolabelling on HEK293T cells fixed in methanol. Images represent negative controls for the pictures showed in **(A)** Fig. 1A, **(B)** Fig. 1B, **(C)** Fig 1C. Nuclei were counterstained with DAPI (blue) and transmission bright-field (BF) images are showing the cell morphology. Scale bar: 10 μm.

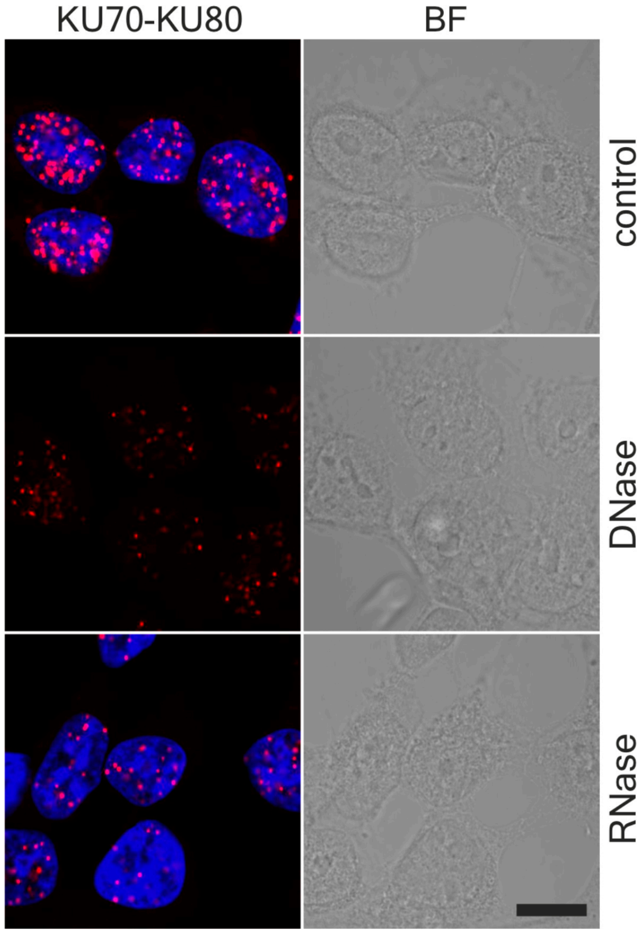

Figure S5. Positive control for *in situ* PLA on HEK293T cells. KU70 and KU80 interactions (red dots) are visualised in the control group and after DNase or RNase digestion. Most of the interactions were localized in the nucleus but some also presented in the cytoplasm. Cells were fixed in methanol. Nuclei were counterstained with DAPI (blue) and transmission bright-field images (BF) showing the cell morphology. Scale bar: 10 µm.

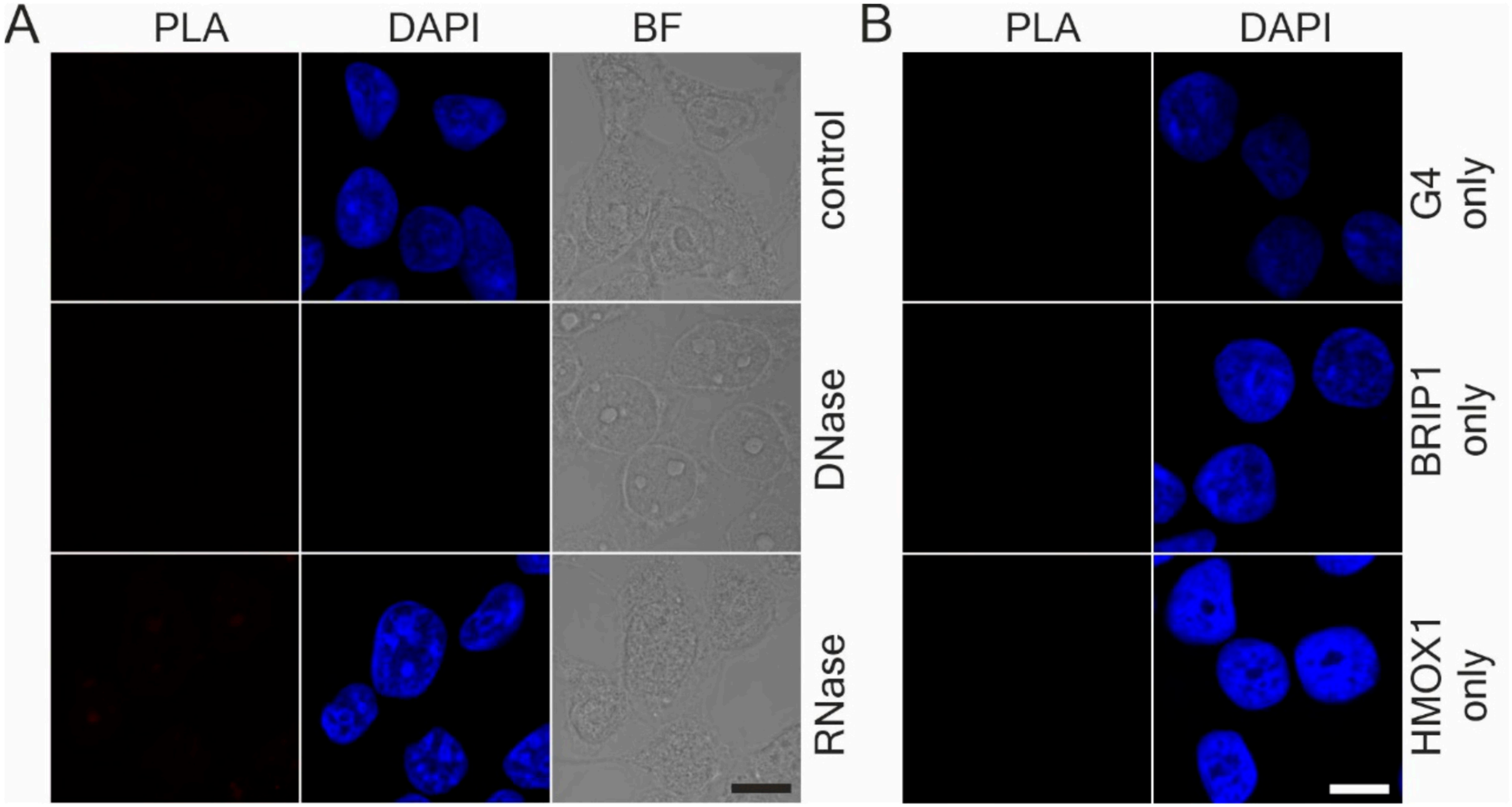

Figure S6. Negative controls for *in situ* PLA on HEK293T cells. (A) Controls with no primary antibodies used for control group and for cells after DNA or RNA digestion. **(B)** Controls with only one primary antibody used for PLA. Cells were fixed in methanol. Cell nuclei were counterstained with DAPI (blue channel) and transmission bright-field images (BF) showing the cell morphology. Scale bar: 10 µm.

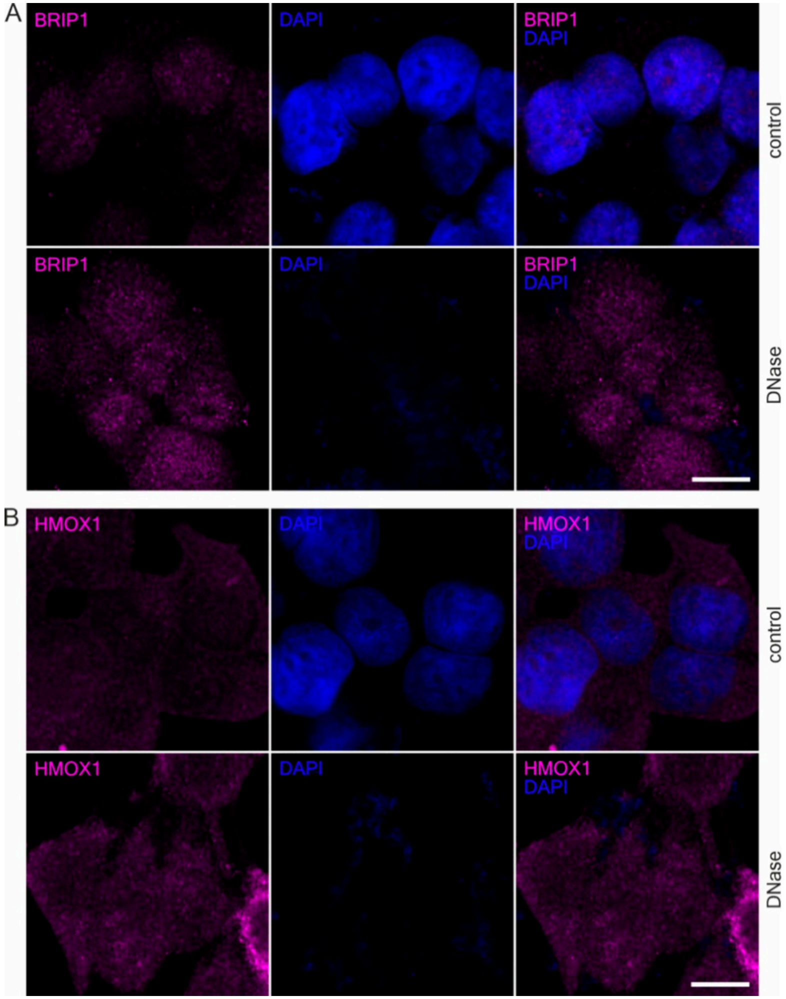

Figure S7. Immunolabelling of BRIP1 and HMOX1 proteins on HEK293T cells. (A) BRIP1 and **(B)** HMOX1 were stained (magenta) in control group and after DNA digestion. Cells were fixed in methanol and nuclei were counterstained with DAPI (blue). Scale bar: 10 μm.

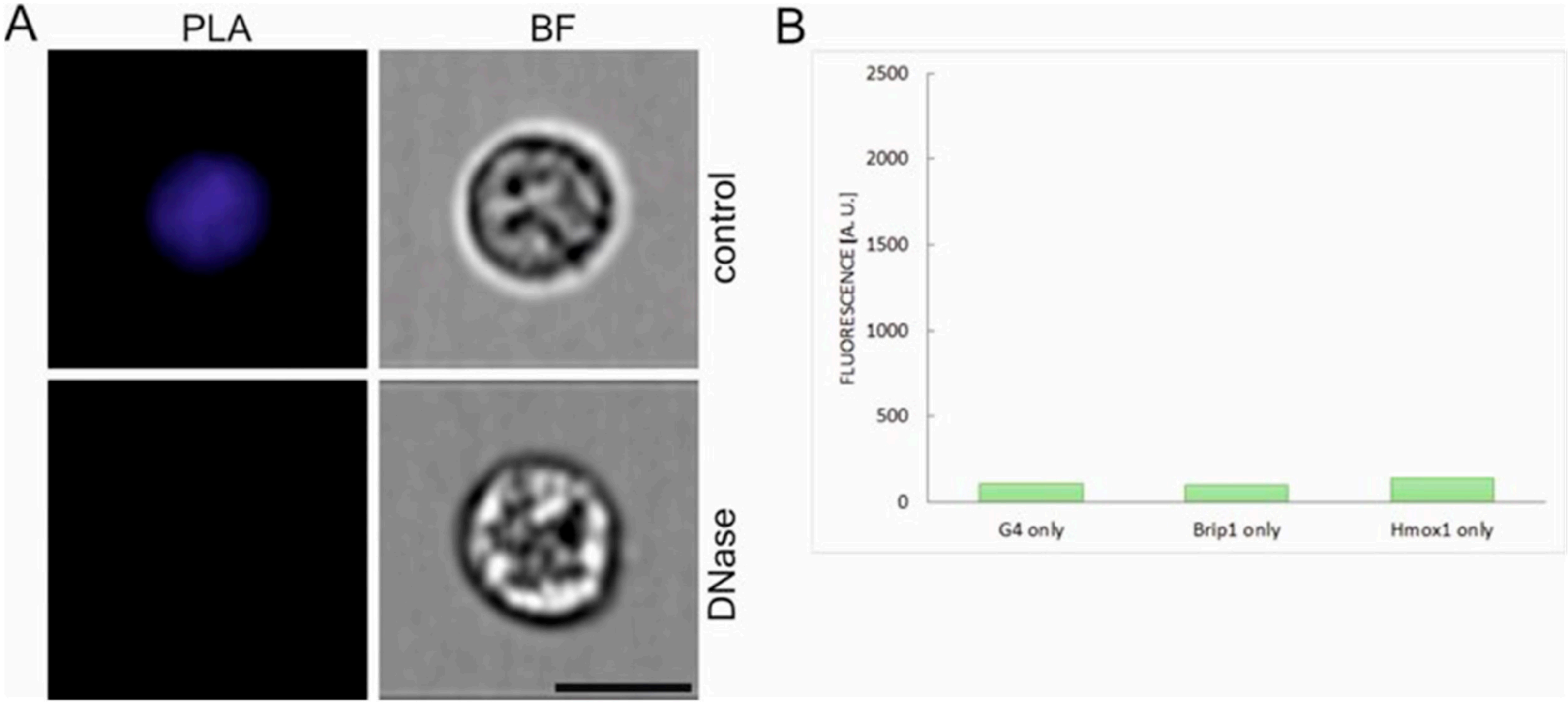

Figure S8. Detection and quantification of PLA signal at a single-cell level in HEK293T cells using flow cytometry – negative controls. Scale bar: 5 µm.

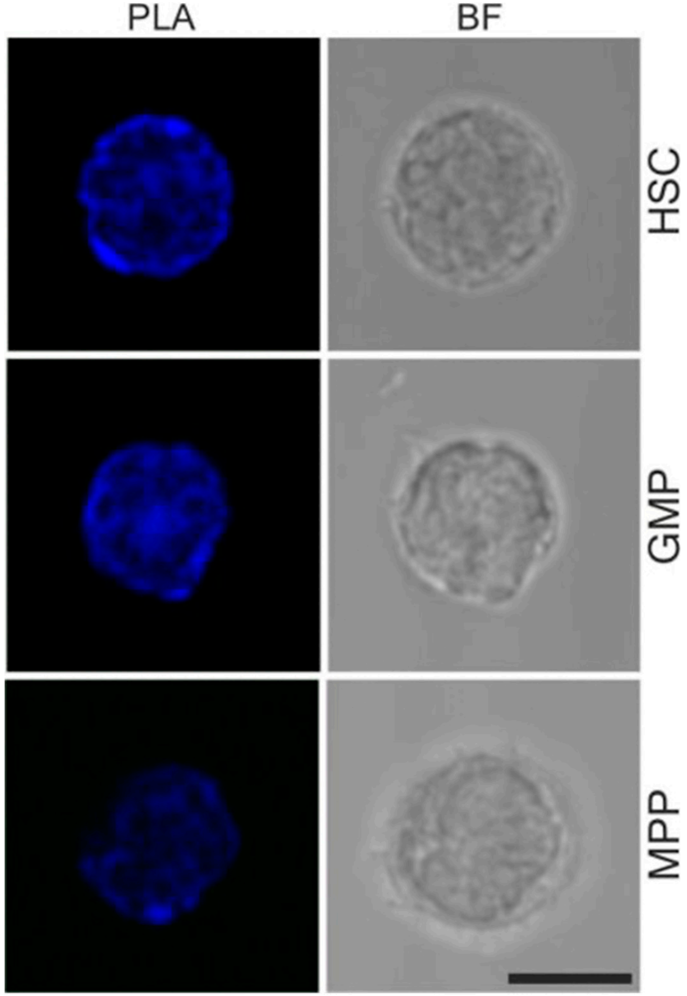

Figure S9. Detection of PLA signal in sorted hematopoietic stem cells – negative controls. Scale bar: 5 µm.

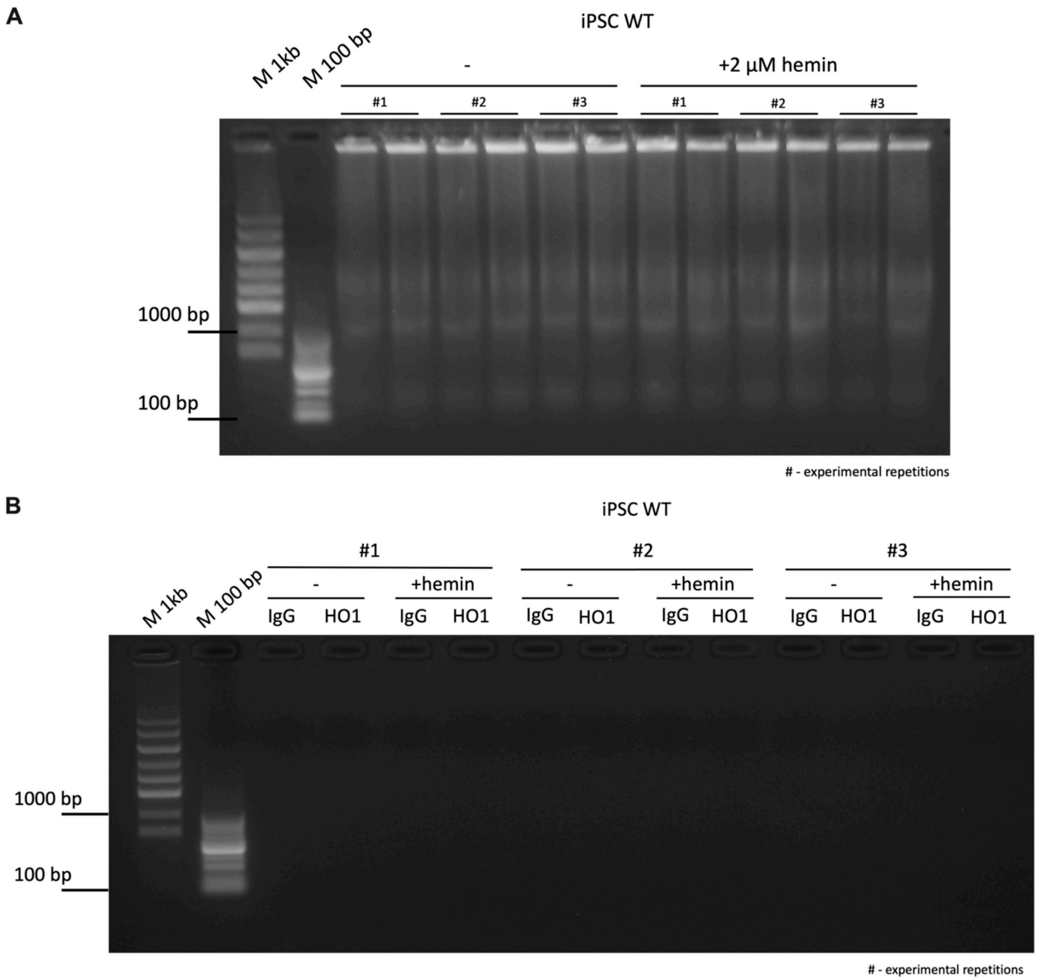

Figure S10. HMOX1 does not bind directly to DNA. (A) Input step of ChIP protocol; chromatin isolated from iPSC *Hmox1*$^{+/+}$ treated (or not) with 2µM hemin and incubate for 4h. n=3 **(B)** Agarose gel after immunoprecipitation, reversal of cross-links and DNA purification.

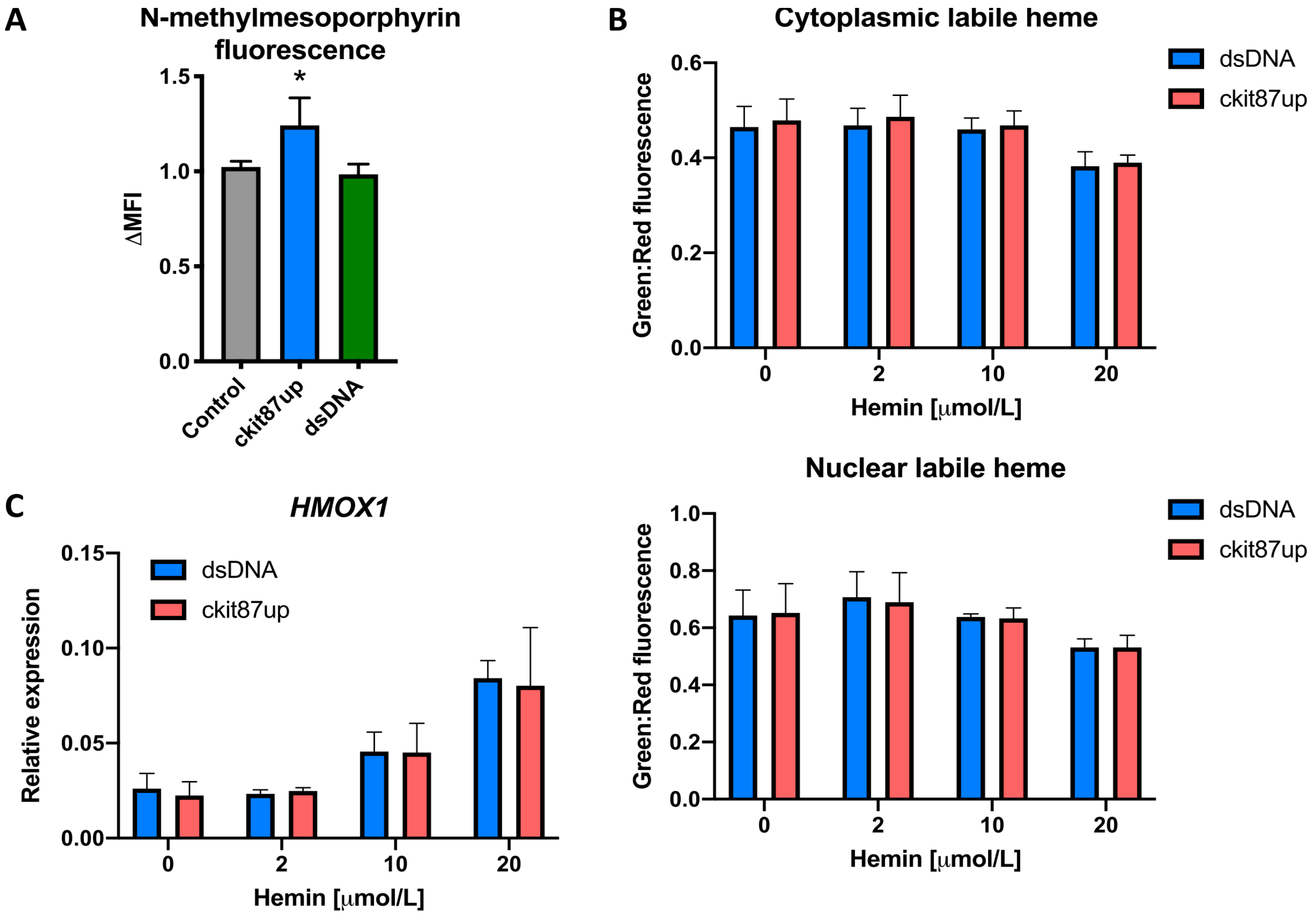

Figure S11. G-quadruplex oligonucleotides do not affect *Hmox1* expression and free heme levels in HEK293T cells. (A) HEK293 cells transfected with 18 nmol\L ckit87up oligonucleotides show enhanced N-methylmesoporphyrin IX fluorescence, N=3, **p*<0.05, One-Way ANOVA with Bonferroni *post hoc t*est **(B)** Ratio of green (eGFP) to red (mKATE2) fluorescence of cytoplasmic and nuclear heme sensors in HEK293T cells transfected with G4-forming oligonucleotides or control DNA and treated with hemin. Green fluorescence of eGFP is quenched by heme. N=3, bars show average ratio + standard deviation **(C)** Transfection with ckit87up oligonucleotides does not affect *Hmox1* expression in HEK293 cells, n=4, Two-Way ANOVA.
